# A *Toxoplasma gondii* lipoxygenase-like enzyme is necessary for virulence and changes localization associated with the host immune response

**DOI:** 10.1128/mbio.01279-23

**Published:** 2023-08-30

**Authors:** Carlos J. Ramírez-Flores, Billy Joel Erazo Flores, Andrés M. Tibabuzo Perdomo, Katie L. Barnes, Sarah K. Wilson, Carolina Mendoza Cavazos, Laura J. Knoll

**Affiliations:** 1 Department of Medical Microbiology and Immunology, University of Wisconsin-Madison, Madison, Wisconsin, USA; University of California, Irvine, Irvine, California, USA

**Keywords:** immune response, leukocytes, lipoxygenase, cytokines, *Toxoplasma gondii*

## Abstract

While the asexual cycle of *Toxoplasma gondii* can occur in any warm-blooded animal, the sexual cycle is restricted to the feline intestine. We previously determined that because cats lack delta-6-desaturase activity in their intestines, they build up excess linoleic acid, which signals *T. gondii* to undergo sexual development. We hypothesized that *T. gondii* oxygenates linoleic acid to signal sexual development, so we examined the *T. gondii* genome for lipoxygenases-like enzymes (TgLOXL) enzymes. We identified seven TgLOXLs that were at least 100-fold more abundant in the cat intestinal versus the tissue culture tachyzoite stage. Parasites deleted in TgLOXL1 (TgΔLOXL1) had no significant growth differences in tissue culture fibroblast cells. Because the sexual development assay begins with brain cysts, we infected mice with TgΔLOXL1 and were surprised to find that TgΔLOXL1 had reduced virulence. The TgΔLOXL1 parasitemia was reduced by 3 d post-infection and largely cleared by 7 d post-infection. At 3 d post-infection, the cytokines interferon gamma (IFN-γ), IL-6, MCP-1, and TNF-α were significantly reduced in TgΔLOXL1-infected mice, which prompted us to examine TgΔLOXL1 in IFN-γ KO mice. We found that IFN-γ KO mice infected with TgΔLOXL1 succumbed to acute infection with the same kinetics as the parental and complemented strains, suggesting that TgLOXL1 plays a role in the IFN-γ signaling cascade. In tissue culture fibroblasts, TgLOXL1 was localized within the parasite, but in leukocytes from infected mice and activated macrophages, TgLOXL1 was localized within the host cytoplasm. These results suggest that TgLOXL1 changes localization in response to host immune activation.

**IMPORTANCE:**

Lipoxygenases (LOXs) are enzymes that catalyze the deoxygenation of polyunsaturated fatty acids such as linoleic and arachidonic acid. These modifications create signaling molecules that are best characterized for modulating the immune response. Deletion of the first lipoxygenase-like enzyme characterized for *Toxoplasma gondii* (TgLOXL1) generated a less virulent strain, and infected mice showed a decreased immune response. This virulence defect was dependent on the mouse cytokine interferon gamma IFNγ. TgLOXL1 changes location from inside the parasite in tissue culture conditions to vesicular structures within the host immune cells during mouse infection. These results suggest that TgLOXL1 plays a role in the modification of the host immune response in mice.

## INTRODUCTION

*Toxoplasma gondii* is one of the most successful parasites in the world, with around 30% of the world’s human population infected (1). Humans are infected congenitally or by consuming tissue cysts or oocysts. The tissue cysts form during the asexual cycle in the muscle and brain of infected animals, whereas oocysts are formed during the sexual cycle in felines and are shed in the feces. In healthy human hosts, the infection can be asymptomatic due to the rapid immune response, allowing the parasite to pivot to a chronic infection. However, in immunocompromised individuals, the infection could lead to chorioretinitis, blindness, encephalitis, and even death. During the early stages of infection, chemokines are released by infected enterocytes. This chemokine signaling results in leukocyte recruitment, followed by cytokine production and parasite clearance (2). IL-12 plays an important role in the stimulation of interferon gamma (IFN-γ) from the natural killer and T cells in *T. gondii* infected mice (3). IFN-γ is considered the main mediator of the acute and chronic defense during *T. gondii* infection (4).

The role of oxygenases in controlling immune response has been well studied (5). The main enzymes involved in the production of these signaling molecules are cyclooxygenases (COXs), cytochrome P450, and lipoxygenases (LOXs). These enzymes belong to the oxidase family, and their main function is to transfer molecular oxygen to their substrate (6). Each one plays a central role in lipid metabolism, specifically, the catalysis of polyunsaturated fatty acids that lead to the production of prostaglandins, thromboxanes, leukotrienes, and lipoxins (7).

The effect of host lipoxygenases and cyclooxygenases on *T. gondii* has been examined. It has been shown that inhibition of the host COX-2 enzyme resulted in the decrease of parasite burden in the brain and intraperitoneal macrophages of *Calomys callosus* rodents (8). Similar results were observed in human trophoblasts and villous explants treated with COX-2 inhibitors (9). Host LOXs appear to be involved in the production of anti-inflammatory responses. When mice are exposed to *T. gondii*, the production of a specialized pro-resolving mediator, Lipoxin A_4_ (LXA_4_), increases. Mice deficient in 5-lipoxygenase, which synthesizes LXA_4_, die at the beginning of chronic infection due to uncontrolled inflammation (10, 11). These studies show the importance of lipid mediators in the control of *T. gondii* infection.

The role of oxygenases encoded by the *T. gondii* genome has not been examined. The pathogenic fungus *Aspergillus flavus* encodes a LOX that is essential for sexual development, suggesting that a LOX-derived metabolite activates the pathway (12). *A. flavus* uses linoleic acid (18:2) but not oleic acid (18:1) to signal sexual development. These results are similar to our finding that *T. gondii* uses linoleic but not oleic acid to signal sexual development (13). In the present study, we focused on the functional characterization of a predicted *T. gondii* lipoxygenase-like enzyme TGME49_315970, hereinafter called TgLOXL1. In this work, we describe the unexpected role of TgLOXL1 during mouse infection, the cytokine response from wild-type (WT) and IFNγ KO mice as well as the localization of TgLOXL1 within the parasite and immune cells.

## RESULTS

### TgLOXL1 is a predicted lipoxygenase of *Toxoplasma gondii*

**Fig 1:**
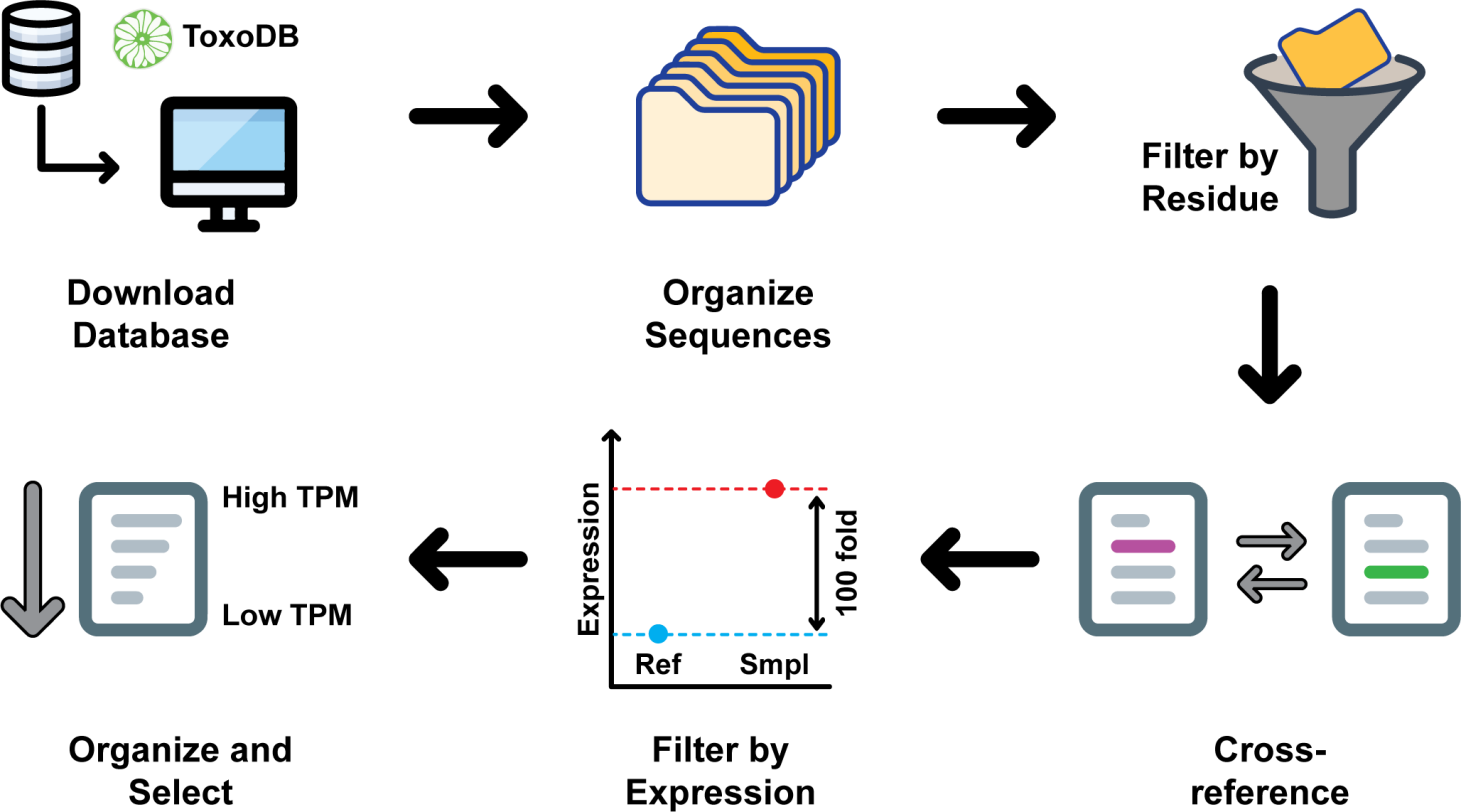
Bioinformatic screening of *T. gondii* proteins to determine potential lipoxygenases. Data were downloaded from ToxoDB and filtered for the presence of an isoleucine or a Valine residue in the C-terminus of the protein. An additional sorting was developed by the selection of 100-fold upregulated proteins during *T. gondii* sexual stage compared to the tachyzoite stage. TGME49_315970 was selected as the potential lipoxygenase of interest due to the high abundance of transcripts during the sexual stage of *T. gondii*.

To study *T. gondii* LOXs potentially involved with sexual development, we performed a bioinformatic screen (Fig. 1). To consider a protein as a LOX of interest we set two parameters: (i) The C-terminus of the protein must be an isoleucine or a valine residue (14, 15), (ii) The transcripts for the LOX should be significantly more abundant in the sexual stages compared to the asexual stages. Our bioinformatic analysis showed that from the 8322 TGME49 annotated proteins from ToxoDB, 35 had a C-terminal isoleucine and 260 had a C-terminal valine residue, a defining characteristic of LOXs. Of the 35 isoleucine candidates, seven were at least 100-fold more abundant in the sexual stage compared to the tachyzoite stage. From the 260 valine candidates, 11 sequences met the same criteria. With the pool of potential candidates greatly reduced (Table 1), TGME_315970 was selected as our protein of interest due to the high abundance of transcripts (1930 TPM) present in the sexual stage of *T. gondii*.

**TABLE 1:** Lipoxygenase candidates

Gene ID	Protein length	Molecular weight	C-term residue	Sexual stage (EES5) unique transcripts (TPM)	Tachyzoite stage unique transcripts (TPM)	Fold change
TGME49_315970	655	69,105	I	1,930	7.67	252
TGME49_218215	584	64,754	I	972	0.28	3,470
TGME49_233360	174	18,324	V	668	5.77	116
TGME49_232170	745	80,968	V	591	0.89	664
TGME49_320290	534	59,292	V	424	0.11	3,850
TGME49_210400	1,354	144,282	V	204	0.05	4,080
TGME49_211360	863	96,500	I	133	0.06	2,210

According to the *T. gondii* transcriptome in feline enteroepithelial stages previously reported (16), the TgLOXL1 (TGME49_315970) transcript is 250-fold more abundant during the enteroepithelial stages compared to the tissue culture tachyzoite stage. Given the potential role of TgLOXL1 in triggering sexual development, we generated a TgLOXL1 depleted strain in PruΔHPT:luciferase parasites. Deletion of the TgLOXL1 gene was confirmed by PCR (Fig. S1A). We also generated complement strains, both untagged and HA-tagged, by random insertion of the TgLOXL1 gene into the ΔTgLOXL1 parasites.

### ΔTgLOXL1 parasites show no distinctive phenotype in tissue culture

The parental PruΔHPT:luciferase, ΔTgLOXL1, or complement untagged parasite were characterized in tissue culture. We saw no replication differences between the parental and ΔTgLOXL1 strains and slight but significant differences between the ΔTgLOXL1 and complement strain at 24 and 36 h. This small difference could be explained by the random insertion of the TgLOXL1 gene in the complemented strain (Fig. S1B). The plaque assays did not show significant changes in plaque size or numbers in human foreskin fibroblast (HFF) monolayers when infected with ΔTgLOXL1 compared to infection with parental strain, but again there was a slight but significant difference between the ΔTgLOXL1 and complement strain (Fig. S1C and D).

### TgLOXL1 is localized within the parasite in HFF cells

**Fig 2:**
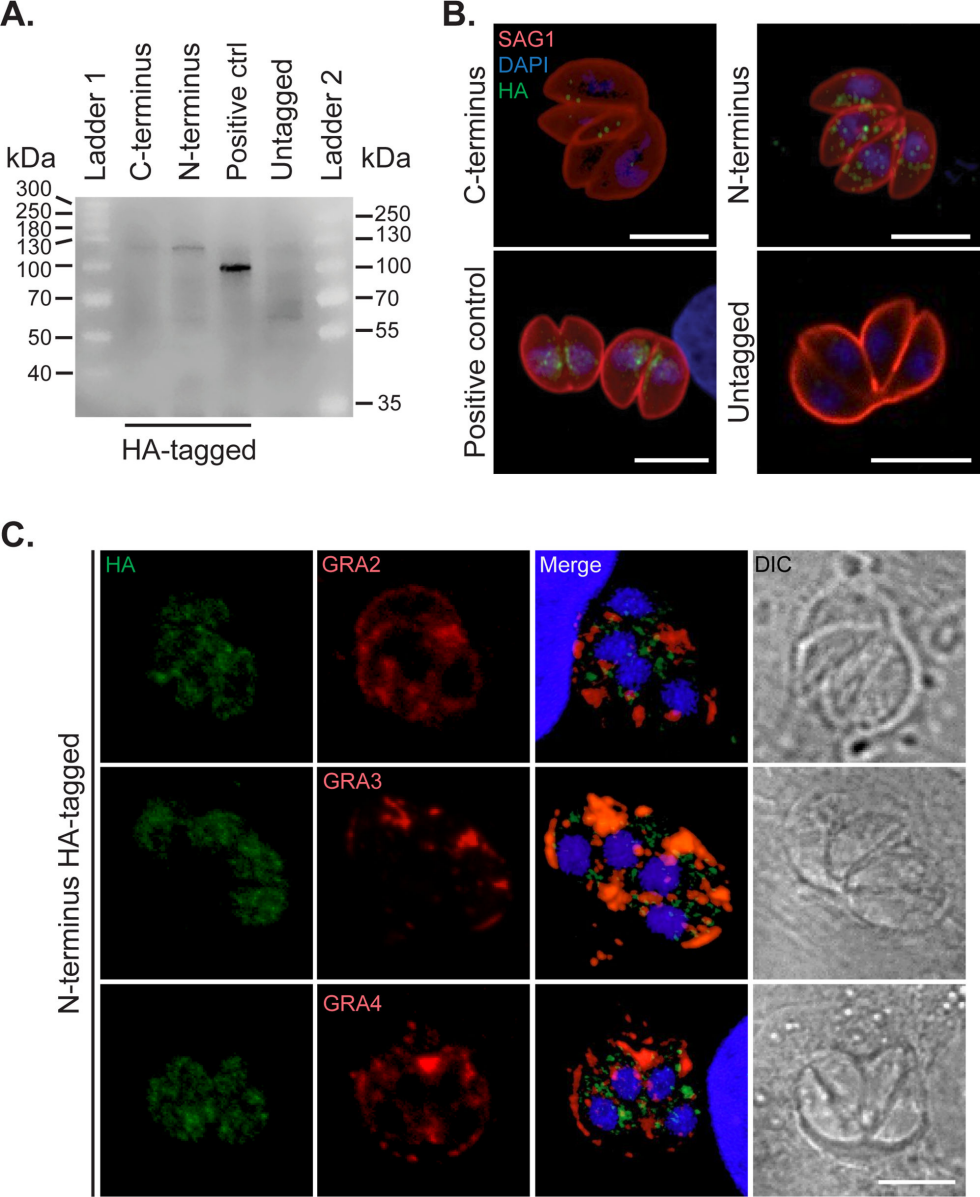
TgLOXL1 is expressed and localized in the cytoplasm of intracellular tachyzoites. (**A**) Parasites expressing HA-tag in the C-terminus or N-terminus were assessed for protein expression by western analysis with anti-HA antibody. To confirm the molecular heights, two protein ladders were used in the same blot: ladder 1 (ThermoFisher Scientific, Cat. 26625) and ladder 2 (Cat. 26619). TgPL1-HA was a positive control, and untagged parasites were a negative control. (**B**) 3D projections of the cytoplasmic distribution of TgLOXL1 (green) and the parasite surface localization of SAG1 (red) in intracellular tachyzoites at 24 h post-infection and visualized by confocal microscopy. TgPL1-HA (green) was a positive control, and untagged parasites were a negative control. (**C**) Colocalization of GRA2, GRA3, or GRA4 (red) and TgLOXL1 (green) in intracellular tachyzoites at 24 h post-infection and visualized by confocal microscopy. Merge shows all the channels in a 3D projection. Nuclei were counterstained with DAPI. Differential interference contrast (DIC) images were taken under the same magnification. All the scale bars are equal to 5 µm.

To localize TgLOXL1, we generated ΔTgLOXL1 complement strains with either an N- or C-terminal HA-tag on TgLOXL1. We opted for tagging both termini to avoid disturbing the LOX activity given that deletion of the C-terminal isoleucine could alter the potential lipoxygenase activity of the enzyme (17). We detected TgLOXL1-HA by western blot (Fig. 2A), so we localized the TgLOXL1 protein in intracellular parasites cultured in HFF cells. Although diffuse labeling was shown with epifluorescence microscopy (Fig. S2), punctate spots within the parasite were detected using a confocal microscope, suggesting a restricted localization of TgLOXL1 (Fig. 2B; Movie S1). Given the distribution of the spots visualized within the parasite, we tested colocalization of TgLOXL1 with dense granule and microneme markers. At 24 h post-infection, TgLOXL1 with either a C- or N-terminal HA-tag showed an intracellular localization that did not colocalize with the dense granule proteins GRA2, GRA3, or GRA4 nor with the microneme protein MIC2 (Fig. 2C; Fig. S2). The HA-tagged TgPL1 protein (18) was used as a positive control for the HA antibody. It is worth noting that we detected the same distribution of TgLOXL1-HA in both C- and N-terminus parasites; however, the abundance of the C-terminal tagged protein was less (Fig. 2A), perhaps due to the HA-tag interference with the catalytic core and destabilization of the protein (Fig. 2B). Hereafter, unless otherwise specified, we preferentially used N-terminal HA-tagged parasites for TgLOXL1 localization.

### ΔTgLOXL1 parasitemia is reduced by chronic infection

We evaluated the role of TgLOXL1 during acute and chronic infection in mice. Because all strains contained the luciferase gene, we estimated the chronic infection parasitemia using luciferase activity. NMRI mice were infected intraperitoneally (i.p.) with 1 × 10^4^ parasites, and parasitemia was measured at 28 d post-infection. According to the luminescence assay, parasites were found in the brains of the infected mice with parental and untagged complement strains, but no parasites were found in ΔTgLOXL1-infected mice (Fig. S3A). We then examined acute infection in NMRI mice with a higher 1 × 10^5^ parasite dose and found that only the ΔTgLOXL1-infected mice survive chronic infection (Fig. S3B). The brains of these NMRI ΔTgLOXL1-infected mice were then stained for *Dolichos Biflorus* Agglutinin (DBA) to visualize the *T. gondii* cyst wall. Only two cysts were counted in the sections from the ΔTgLOXL1-infected mice, highlighting that there were less than 100 cysts in the entire brain, but the cysts that were seen had intact walls (Fig. S3C). We then infected Swiss Webster mice with 1 × 10^4^ parasites of each strain and monitored by *in vivo* imaging system (IVIS) at 28 d post-infection. We saw again a significant reduction in the relative light units during chronic infection in the ΔTgLOXL1-infected Swiss Webster mice (Fig. S3D).

### ΔTgLOXL1 parasites are cleared in C57BL/6 mice by day 7 post-infection

**Fig 3:**
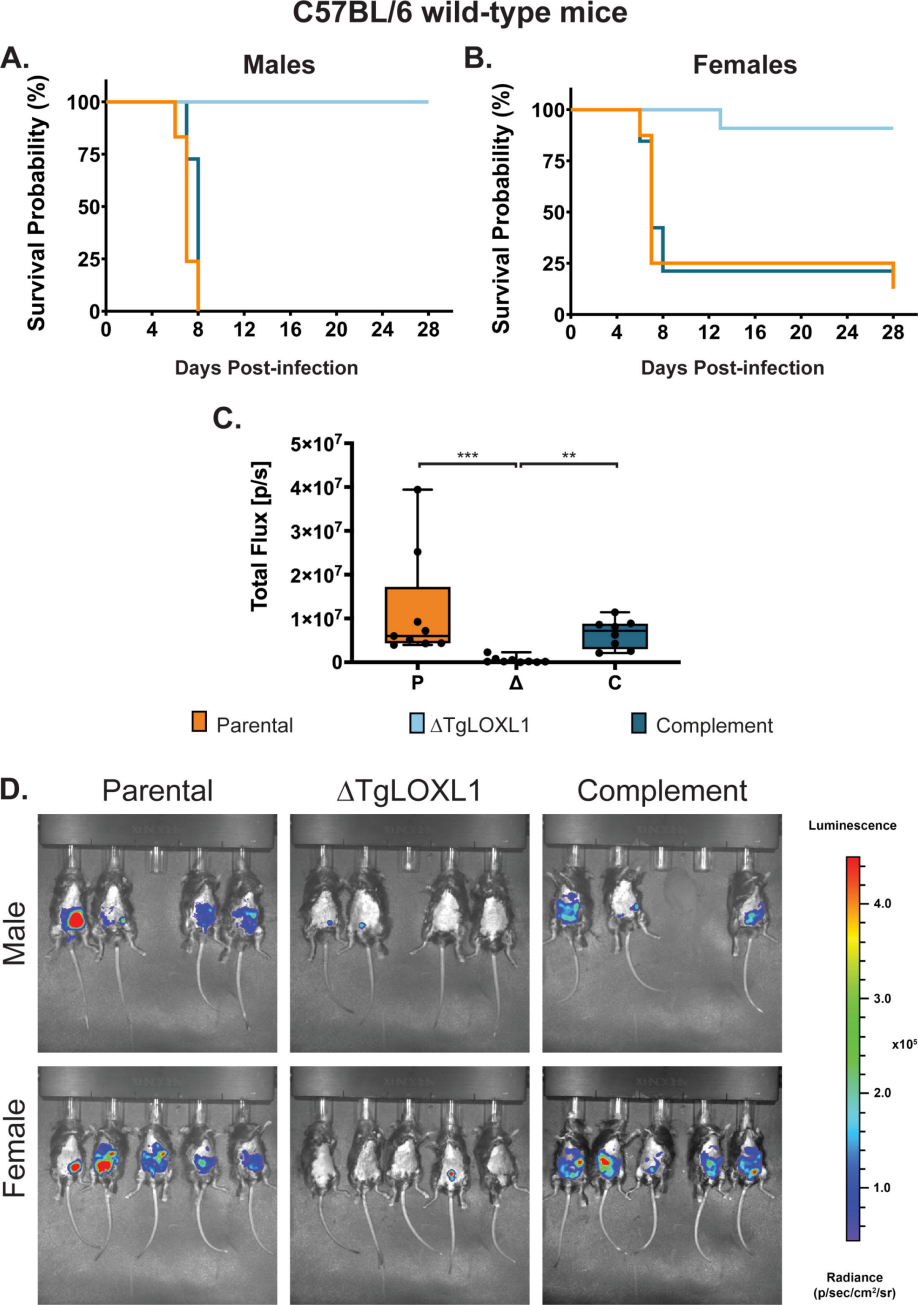
ΔTgLOXL1 parasites are avirulent in wild-type mice. Shown is a combination of two independent experiments of 3–5 C57BL/6 wild-type mice males (**A**) or all females (**B**), with a total of 6–9 mice per strain. Mice were i.p. infected with 1 × 10^5^ of each strain, and health was monitored up to 28 d post-infection. (**C and D**) Females and males C57BL/6 WT were infected i.p. with 1 × 10^4^ parasites of each strain, and 7 d post-infection they were imaged ventrally by IVIS. (**C**) Shown is the total flux obtained by measuring the luminescence intensity in the peritoneal cavity of mice. (**D**) Shown are the images for each strain and gender separately. For all mice, the abdominal hair was removed to avoid signal interference, and the exposure time was the same.

We evaluated the role of TgLOXL1 during acute infection in C57BL/6 mice because many immune response deletion strains are available in the C57BL/6 background. Survival curves of C57BL/6 mice infected with 1 × 10^4^ parental parasites showed some lethality during acute infection, but all mice infected with 1 × 10^4^ ΔTgLOXL1 or complement parasites survived (Fig. S3E). We then infected C57BL/6 mice i.p. with 1 × 10^5^ parasites and monitored their health over time. Mice infected with parental or untagged complement strains showed signs of declining health by 7 d post-infection, with most becoming moribund (Fig. 3). Mice infected with the ΔTgLOXL1 strain did not show any symptoms of infection by 28 d post-infection. Interestingly, while all of the male mice infected with parental or complemented strains needed to be sacrificed (Fig. 3A), 20% of C57BL/6 female mice infected with parental or complemented strains recovered and survived out to 28 d post-infection (Fig. 3B).

To track the progression of infection by an *in vivo* imaging system and avoid the moribund state, we infected C57BL/6 WT mice with this reduced dose of 1 × 10^4^ parasites. Mice infected with ΔTgLOXL1 parasites showed no or poor signal restricted to the site of the inoculation at 7 d post-infection; in contrast, mice infected with parental and complement strains showed higher levels of parasitemia along the peritoneal cavity (Fig. 3B and C). No sex-dependent differences in parasitemia were found between the male and female mice. The clearing of the ΔTgLOXL1 parasites by day 7 post-infection explains the lack of symptoms during acute infection and cysts by day 28 post-infection (Fig. S3).

### Mice infected with ΔTgLOXL1 parasites show a reduced cytokine response

**Fig 4:**
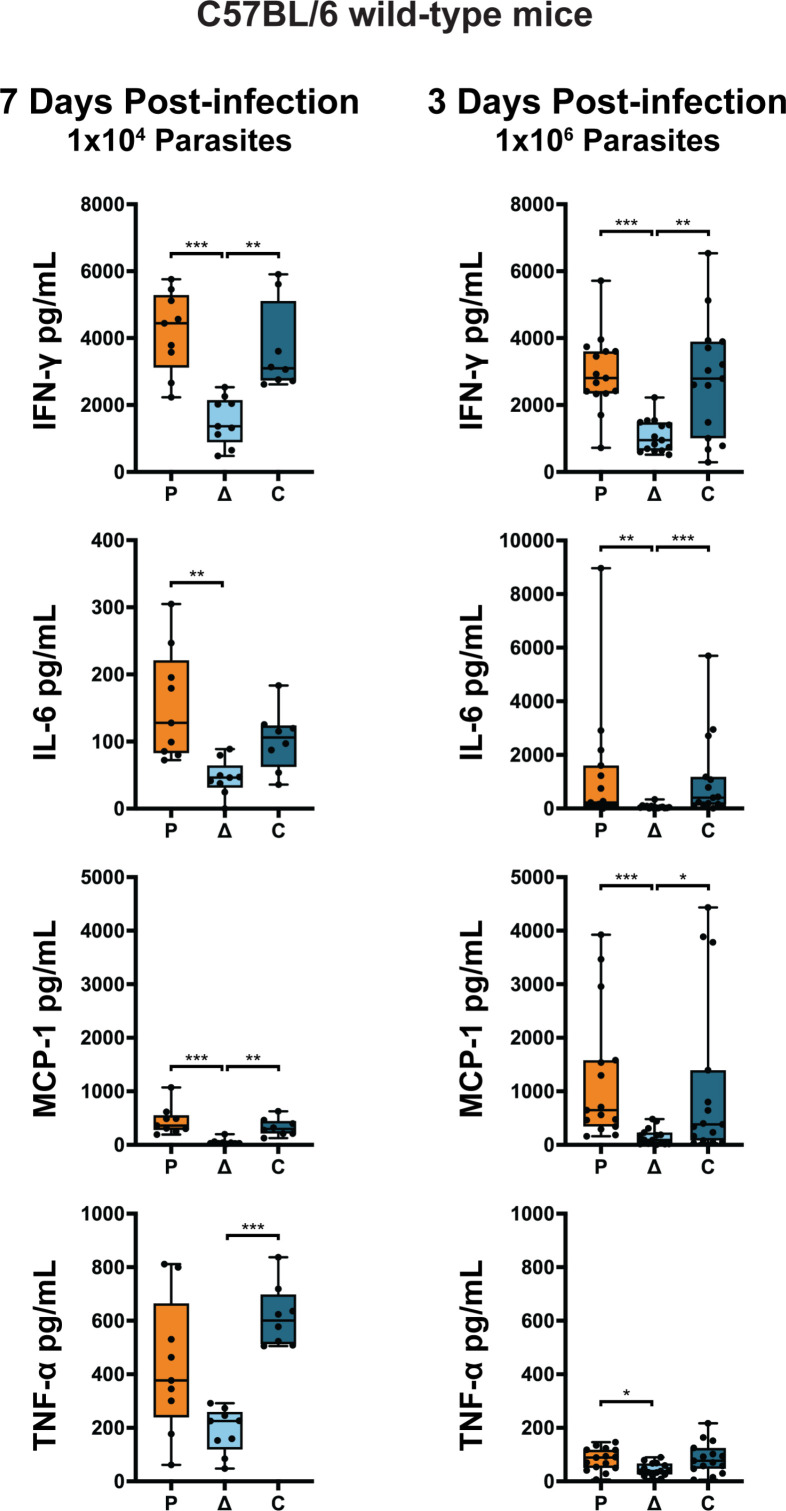
ΔTgLOXL1 parasites produced a reduced cytokine response in wild-type mice. Blood serum of female and male C57BL/6 WT mice was collected to analyze the cytokine response. Mice were infected with 1 × 10^4^ or 1 × 10^6^ parasites of each strain and analyzed at 7 or 3 d post-infection, respectively. IL-10 and IL-12p70 were not detected at either day. The statistics were performed using one-way Analysis of Variance (ANOVA) in the GraphPad Prism software. The *P* value was considered as follows: * <0.05, ** <0.005, and *** <0.0005.

The pronounced phenotype that was observed *in vivo* prompted us to investigate the host immune response mounted against the infection. We infected C57BL/6 WT mice with 1 × 10^4^ parasites of either parental, ΔTgLOXL1, or untagged complement strains and collected sera from tail bleeds: before infection, 3 d post-infection, and a final bleed at 7 d post-infection. Sera were then used to quantify the concentration of inflammatory cytokines (IFN-γ, IL-6, MCP-1, IL-10, IL-12p70, and TNF-α). Similar to the progression of the infection observed by IVIS at day seven post-infection, the cytokine response was higher in those mice infected with parental and complement parasites, with no differences between those two groups (Fig. 4). The immune response in ΔTgLOXL1-infected mice was significantly reduced compared to the parental and complement strains in all the cytokines tested (Fig. 4). With an infection of 1 × 10^4^ parasites, the cytokines at 3 d post-infection were in low abundance and were not able to be detected (data not shown).

To determine the cytokine response during the early infection, we challenged the mice with a larger dose (1 × 10^6^ parasites) of each strain and measured the cytokines at 3 d post-infection. As expected from a higher parasite load, the levels of cytokines in the sera increased proportionally. At 3 d post-infection, all the cytokine levels of IL-6, MCP-1, TNF-α, and IFN-γ in ΔTgLOXL1-infected mice were lower compared to the cytokine level in parental-infected mice (Fig. 4). These results are consistent with the IVIS data, showing that even with this large dose, some mice were able to clear the infection when infected with ΔTgLOXL1 parasites, by 3 d post-infection (Fig. S4). However, the avirulent phenotype of ΔTgLOXL1 is dosage dependent as mice infected with 1 × 10^8^ ΔTgLOXL1 parasites were symptomatic and moribund by 11 d post-infection (data not shown). These results suggest that TgLOXL1 can modulate immune response as early as 3 d post-infection.

### IFN-γ KO mice are susceptible to ΔTgLOXL1 parasites

**Fig 5:**
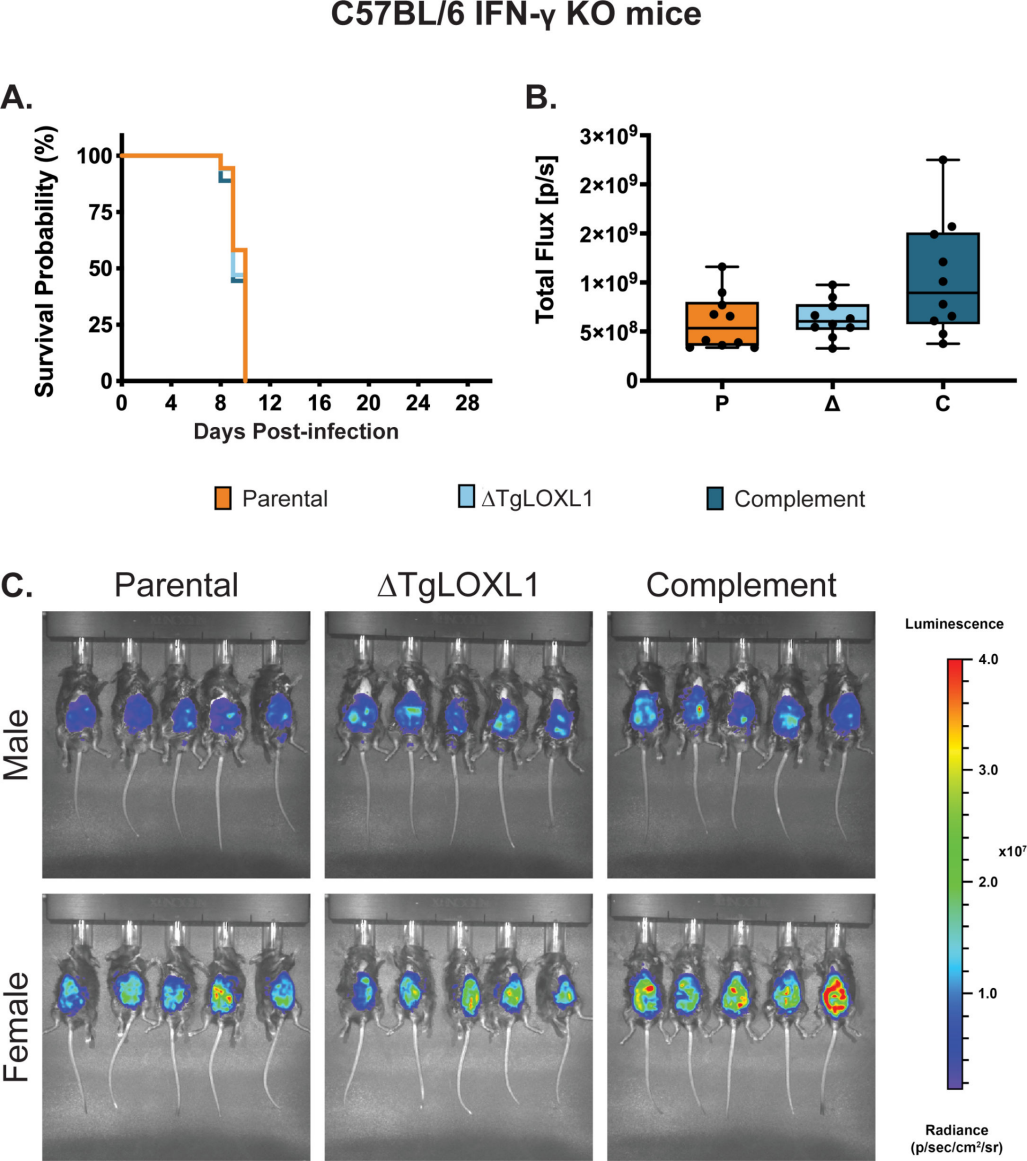
ΔTgLOXL1 parasites are virulent in IFN-γ KO mice. (**A**) Shown is a combination of two independent experiments of 2–5 IFN-γ KO mice, either all males or all females, with a total of 7 mice per strain. Mice were i.p. infected with 1 × 10^4^ of each strain, and their health was monitored until they were moribund. (**B and C**) Females and males IFN-γ KO were infected i.p. with 1 × 10^3^ luciferase-expressing parasites of each strain, and 7 d post-infection they were imaged ventrally by IVIS. (**B**) Shown is the total flux obtained by measuring the luminescence intensity in the peritoneal cavity of mice. (**C**) Shown are the images for each strain and gender separately. For all mice, the abdominal hair was removed to avoid signal interference, and the exposure time was the same.

The IFNγ response is critical for the host defense against *T. gondii* (4). IFN-γ levels were significantly lower when mice were infected with ΔTgLOXL1 (Fig. 4), so we tested if the immune response modulation by TgLOXL1 was IFN-γ dependent by infecting IFN-γ KO mice. Male and female IFN-γ KO mice were infected with 1 × 10^4^ parasites of parental, ΔTgLOXL1, and complement parasites. All infected mice needed to be sacrificed on days 9–10 (Fig. 5A), making it impossible to obtain cysts in their brains, which requires an incubation period of at least 21 d. We repeated the infection with a lower parasite burden of 1,000 parasites and then with 100 parasites. In both cases, the spread of the infection was such that all mice died by day 10 post-infection (data not shown). At 7 d post-infection, all of the IFN-γ KO mice infected with 1 × 10^3^ parental, ΔTgLOXL1, or complement strains showed similar parasitemia (Fig. 5B and C). These results suggest that the ΔTgLOXL1 parasites can invade and generate acute infection efficiently in the absence of IFN-γ.

We analyzed the cytokine response in IFN-γ KO mice at 7 d post-infection. The results showed that in the absence of IFN-γ, the concentration of MCP-1 increased ~10-fold, and IL-6 increased ~100-fold when compared to the C57BL/6 WT mice, suggesting that other cytokines are upregulated to try to control parasitemia (Fig. S5). It is interesting that TNF-α is significantly lower in ΔTgLOXL1-infected mice compared to parental- and complement-infected WT mice, but TNF-α is significantly higher in ΔTgLOXL1-infected mice compared to parental- and complement-infected IFN-γ KO mice. These differences are clearly not protecting IFN-γ KO mice against *T. gondii* infection as the mice succumb to infection with the same kinetics regardless of which strains they were infected with.

### TgLOXL1 is localized in the cytoplasm of infected leukocytes

**Fig 6:**
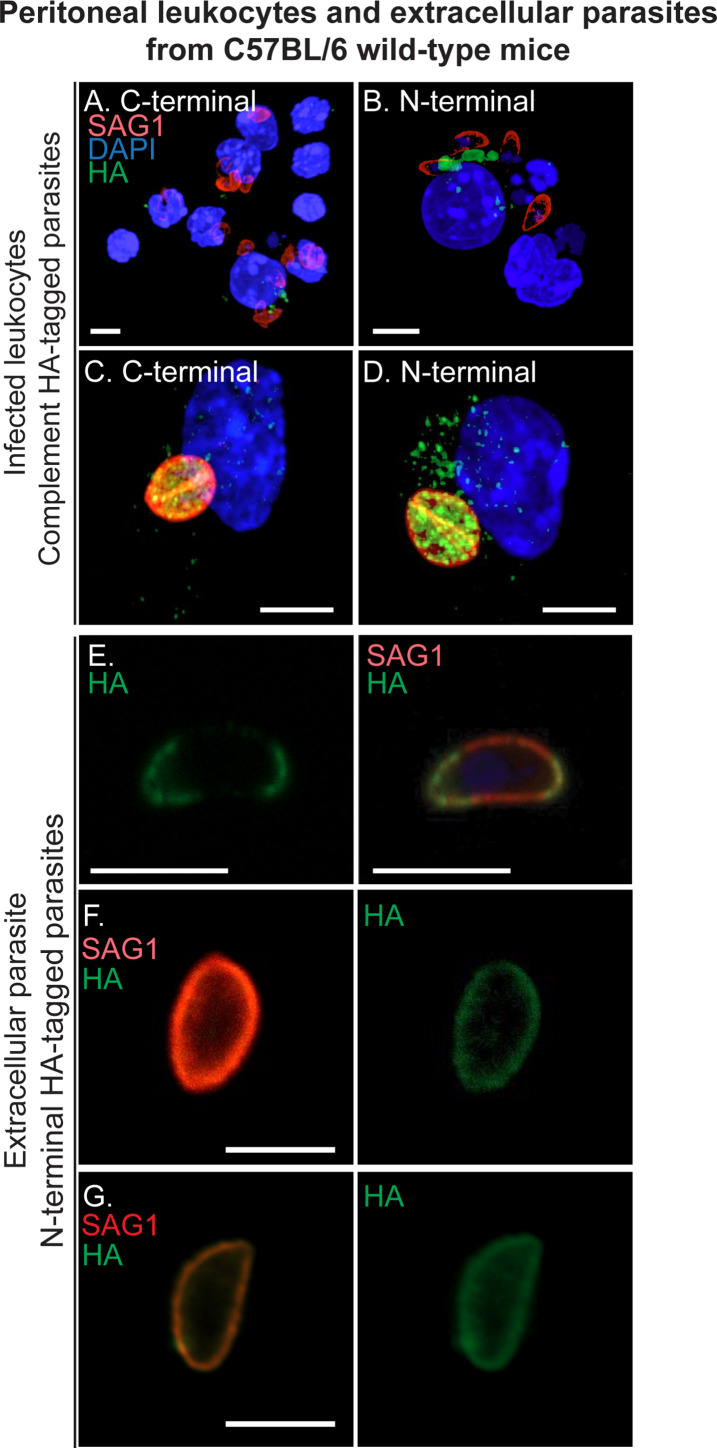
TgLOXL1 is released by the parasite inside infected leukocytes. 3D projections of infected leukocytes isolated from the peritoneal cavity of infected mice. Mice were infected with 2 × 10^6^ C-terminal HA-tag (**A and C**) or N-terminal HA-tag parasites (**B and D**), and 3 d post-infection leukocytes were isolated. Parasites were stained for SAG1 (red) and TgLOXL1-HA (green), and nuclei were counterstained with DAPI. (**E–G**) Examples of extracellular parasites isolated from the peritoneal cavity of infected mice with N-terminal HA-tagged parasites. TgLOXL1-HA (green) is distributed on the parasite membrane partially co-localizing with SAG1 (red). Nuclei were counterstained with DAPI. All the scale bars are equal to 5 µm.

During pathogen infections in mammals, it is well known that lipoxygenases and their products play an important role in modifying the host immune response by promoting proinflammatory or anti-inflammatory processes (19). To explore the role TgLOXL1 plays during animal infection, we examined TgLOXL1-HA localization within infected leukocytes. We infected mice with the C- and N- terminal HA-tagged parasites, and at 3 d post-infection, we collected the infected peritoneal leukocytes. We found two populations of infected peritoneal leukocytes: (i) leukocytes containing a single parasite per vacuole and the TgLOXL1-HA protein is no longer localized within the parasite but instead found within vesicle-like structures within the cytoplasm of leukocytes in both complemented strains (Fig. 6A and B; Movie S2) and (ii) leukocytes containing at least two parasite per vacuole and the TgLOXL1-HA within the parasite as well as within the host cytoplasm (Fig. 6C and D; Movie S3). In extracellular parasites isolated from the peritoneal cavity, TgLOXL1 is localized on the surface of the parasite (Fig. 6E through G). While 100% of TgLOXL1-HA is distributed on the plasma membrane of the parasite in extracellular parasites from the peritoneal cavity, some parasites show patches of TgLOXL1-HA (Fig. 6E) and some show continuous distribution, co-localizing with SAG1 (Fig. 6F and G). In contrast, TgLOXL1-HA was not detected in extracellular parasites isolated from infected HFF cells (data not shown). Of note, no staining of the TgLOXL1 signal was detected in uninfected leukocytes in the same samples (data not shown).

**Fig 7:**
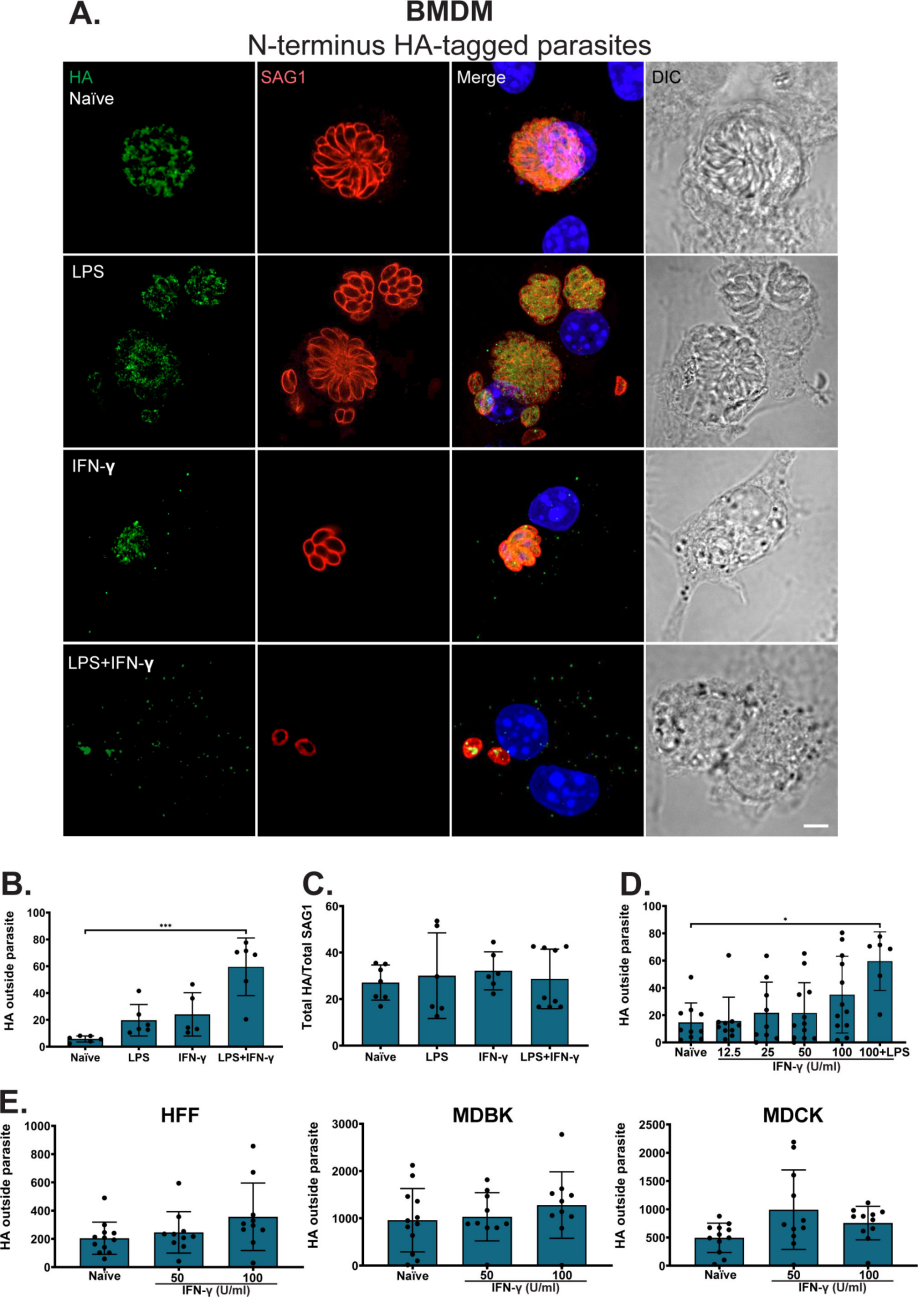
Releasing of TgLOXL1 is reproducible in BMDMs by stimulation with LPS and IFN-γ. (**A**) Shown is a representative of the infected then activated BMDMs in cell culture. BMDMs were infected with N-terminal HA-tagged parasites for 3 h, then stimulated with 100  ng/mL LPS, 100 U/mL IFN-γ or 100 U/mL IFN-γ, and 100  ng/mL LPS, and then imaged after 48 h. Naïve BMDMs were used as control. Parasites were stained for SAG1 (red) and TgLOXL1-HA (green), and nuclei were counterstained with DAPI. All scale bars are equal to 5 µm. (**B**) Shown is TgLOXL1-HA released by the parasite to the cytoplasm of BMDMs by quantification of cytoplasmic TgLOXL1-HA (host) and normalized against cytoplasmic TgLOXL1-HA (parasite). (**C**) Shown is total TgLOXL1-HA by quantification of total fluorescence of HA and normalized against SAG1. (**D**) Shown is TgLOXL1-HA released by the parasite to the cytoplasm of BMDMs by quantification of cytoplasmic TgLOXL1-HA (host) and normalized against cytoplasmic TgLOXL1-HA (parasite). (**E**) Shown is TgLOXL1-HA released by the parasite to the cytoplasm of cell lines by quantification of cytoplasmic TgLOXL1-HA (host) and normalized against cytoplasmic TgLOXL1-HA (parasite). The statistics were performed using one-way ANOVA in the GraphPad Prism software. The *P* value was considered as *** <0.0005.

The fact that TgLOXL1 was not detected within infected HFF but within vesicle-like structures within the cytoplasm of leukocytes isolated from mice prompted us to examine if we could recapitulate this localization change in tissue culture immune cells. We isolated bone marrow-derived macrophages (BMDMs), infected them with HA-tagged parasites, and then stimulated them with lipopolysaccharide (LPS), IFN-γ, or both. After 48 h of stimulation with LPS or IFN-γ, intracellular parasites showed a TgLOXL1 localization within the parasite as well as in the host cytoplasm (Fig. 7A). When infected BMDMs were stimulated with both LPS and IFN-γ, the amount of TgLOXL1-HA released into the host cytoplasm seemed to increase compared to BMDMs stimulated with either LPS or IFN-γ alone (Fig. 7A). In naïve BMDMs, TgLOXL1 was only localized within the parasite, and it was not detected in the host cytoplasm (Fig. 7A). These results were consistent with what we saw in immune cells isolated from mice (Fig. 6A through D).

In the BMDMs activated with LPS and IFN-γ, the majority of the TgLOXL1 seemed to be within the host cell cytoplasm. To quantify this localization change, we measured the total fluorescence of TgLOXL1-HA within the host cytoplasm under different activation conditions. Sixty percent of TgLOXL1-HA was localized in the host cytoplasm when BMDMs were stimulated with both LPS and IFN-γ, whereas only 20% of TgLOXL1-HA was localized in the host cytoplasm when BMDMs were stimulated with either LPS or IFN-γ (Fig. 7B). We found that the total TgLOXL1-HA fluorescence did not change upon any stimulation condition compared to the naïve BMDMs (Fig. 7C), highlighting that the protein is unlikely to be upregulated with stimulation. As a control, no staining of TgLOXL1-HA was detected in uninfected BMDM or in BMDM infected with untagged parasites (Fig. S6A and B). BMDM stimulation with 100 ng/mL LPS and 100 U/mL IFN-γ was required for significant release of TgLOXL1 compared to various concentrations of IFN-γ alone (Fig. 7D). To evaluate if TgLOXL1 is a *T. gondii* effector counteracting host IFN-γ, we tested its secretion in other cell types IFN-γ-stimulated (Fig. 7E). Our results showed no differences in the secretion of TgLOXL1 in HFF, MDCK, or MDBK cells stimulated with 50 or 100 U/mL IFN-γ compared to naïve cells.

To confirm that TgLOXL1 is released into the host cytoplasm and not localized in the host due to a compromised parasitophorous vacuole membrane (PVM), we used a differential permeabilization protocol. Infected and activated BMDMs were fixed, treated with 0.005% saponin, and then stained for SAG1. This low percentage of saponin was found to permeabilize the host plasma membrane but not the PVM (20). After saponin treatment, only extracellular parasites were stained with SAG1, indicating an intact PVM (Fig. S6). We confirmed these results by staining luciferase in triton x-100 permeabilized BMDMs. Under triton condition, we found luciferase in the parasite cytoplasm but not in the host cytoplasm, indicating that the parasites are viable and not leaking cytoplasmic content into the PVM during the stimulation (Fig. S6C). All together, these results suggest that the activation of BMDMs with both LPS and IFN-γ is required to reproduce the results of our murine model. The fact that TgLOXL1 is localized in vesicles within the leukocytes was interesting especially because TgLOXL1 is not predicted to contain a signal peptide. Perhaps this localization change is important for parasite survival and/or manipulation of the host immune response.

### TgLOXL1 and its role in lipid metabolism

To test the role of TgLOXL1 in lipid metabolism, we treated BMDMs with the COX-2 inhibitor, Meloxicam. It was previously reported that Meloxicam, a COX-2 inhibitor, inhibits *T. gondii* intracellular proliferation in peritoneal macrophages at a concentration of 250–1,000 µg/mL and upregulates the immune response in *T. gondii*-infected *C. callosus* (8). When we treated BMDMs with 500 µg/mL Meloxicam, we found a detrimental effect on the macrophages which included cell detachment insinuating cell death in uninfected and unstimulated BMDMs (data not shown). We then pretreated mice with Meloxicam, left them uninfected or infected with parental, ΔTgLOXL1, and complemented strains, and continued treatments for 7 d. We measured the serum cytokine response 7 d post-infection and saw a decrease in TNF-α and IFN-γ in Meloxicam-treated mice infected with parental strain compared to untreated. We did not see differences in the cytokine response of TNF-α or IFN-γ in mice infected with ΔTgLOXL1 parasites with or without Meloxicam treatment (Fig. S6G). We did not find any difference in MCP-1, IL-10, IL-6, and IL-12 abundance between treatments or strains (data not shown). The fact that we also saw a decreased TNF-α in ΔTgLOXL1 parasites could support the hypothesis that TgLOXL1 plays a role in the IFN-γ cascade and not on IFN-γ directly. The role of other lipid mediators such as celecoxib or etoricoxib and their association with TgLOXL1 is an avenue that we will explore in the future.

## DISCUSSION

Our laboratory has become interested in the lipoxygenases of *T. gondii* because of their potential role in modifying linoleic acid into lipid mediators that trigger sexual development (13). Our bioinformatic search for potential enzymes involved in processing linoleic acid led us to TgLOXL1, a protein with sequence features like those found in lipoxygenases. In addition to the structural motifs, RNASeq data showed that this gene was highly transcribed in the sexual stages of *T. gondii* in contrast to the tachyzoite stage. Tissue culture characterization of ΔTgLOXL1 did not reveal any phenotypes in fibroblast cells, as we had expected from a protein that was negligibly transcribed in the tachyzoite stage. However, the ΔTgLOXL1 did have a deleterious phenotype during animal infection. When C57BL/6 mice were infected with ΔTgLOXL1, the mice were able to clear the infection by 7 d post-infection. While this result was disappointing for the analysis of TgLOXL1 during sexual development because brain cysts are necessary for our current sexual development assay (13), this virulence defect prompted us to further characterize this protein. We found TgLOXL1 to be present in the cytoplasm of parasites *in vitro*, but this changed once the parasite was injected via i.p. into C57BL/6 WT mice. *In vivo*, TgLOXL1 was present in the membrane of extracellular parasites, and more interestingly, the protein appeared to be within vesicles of the infected host immune cell.

Considering our observations, we wondered how a non-essential protein for the parasite could present such a strong phenotype *in vivo*. We believe the answer is in the fundamental biology of the parasite’s infection mechanisms. When *T. gondii* invades, it uses three particular organelles in the apical complex in a coordinated fashion, micronemes, rhoptries, and dense granules (21). Microneme proteins help in the initial attachment and trigger proteins in the rhoptries to be released to help the parasite avoid detection by the immune system. Once inside the cell, proteins from the dense granules contribute to the hijacking of the host cell and pave the way for the parasite to start replication (21, 22). In the host cell, IFN-γ pathways are known as the first line of defense against *T. gondii*, and the most studied to date is the JAK-STAT pathway (4, 23). ROP16 has been shown to interfere with STAT1 transcripts, altering the regulation of genes related to IFN-γ and IL-12 *in vitro* (24, 25). There are three main ways in which IFN-γ limits parasite replication. First by induction of Reactive Oxygen Species and Reactive Nitrogen Species to combat intracellular parasites (26, 27). Second, induction of Indoleamine 2,3-dioxygenases (IDO1 and IDO2) to degrade tryptophan and restrict nutrients used by the parasite (28 – 31). Third, activation of Immunity Related GTPases (IRGs) that disrupt the parasitophorous vacuole membrane and clear the parasite from the cell (32, 33). Thus, disrupting IFN-γ mediated pathways is crucial for parasite survival.

From the protein effectors that *T. gondii* uses to evade the immune response, we will focus on ROP and GRA proteins given their similarities to the behavior of TgLOXL1 both *in vitro* and *in vivo*. In mice, the first line of attack against IFN-γ mediated signals comes from ROP 5, 17, and 18 working in synergy to block IRGs (33, 34). Later in the infection, a GRA protein (TgIST) forms a high molecular weight complex inhibiting the STAT1 pathway that helps clear the parasite (25, 35, 36). Both classes of proteins seem to be non-essential *in vitro* but are important *in vivo* due to the interplay between them and the host’s immune response (37 – 39). This behavior matches what we observe in TgLOXL1, of particular note is ROP38 which is highly upregulated in bradyzoites yet is still deemed non-essential (38, 40).

In our experiments, wild-type mice infected with 1 × 10^6^ ΔTgLOXL1 parasites started to clear the infection after just 3 d. This rapid clearance resembles what Olias et al. observed when the absence of TgIST led to enhanced clearance and reduced growth of type II parasites (35). This clearance occurred in cells that were infected and then activated with IFN-γ but not in naïve cells that had been prestimulated. These results indicate that TgIST acts prior to IFN-γ signaling by blocking the transcriptional process. We found that there was no clearance of ΔTgLOXL1 in IFN-γ KO mice. This result suggests that the mechanism that TgLOXL1 uses is somehow altering IFN-γ signaling resulting in uncontrolled parasite replication leading to the host’s demise (41, 42).

Here, we found that the localization of TgLOXL1 in the cytoplasm of BMDMs depends on their activation. In naïve BMDMs, TgLOXL1 was localized inside the parasite; however, after the classical activation of BMDMs by LPS and IFN-γ, TgLOXL1 was detected mainly in the cytoplasm of BMDMs. Previous work in our lab highlighted the potential importance of lipid metabolism in manipulating the host immune response by examining a patatin-like protein that protected *T. gondii* from degradation in activated macrophages (18). The absence of the protein, named TgPL1, was correlated with failure to suppress nitric oxide production and degradation in activated macrophages (43). After 33 h in activated macrophages, parasites lacking TgPL1 showed extensive vesiculation and breakdown of the PVM. This patatin-like protein had a punctate cytoplasmic localization but was distinct from acidocalcisomes, micronemes, and rhoptries suggesting that its function is independent of secretory events associated with parasite invasion. However, TgPL1 changes localization from within the parasite to the PV and PVM after macrophage activation, colocalizing with GRA4 (43). Analysis of ΔTgPL1 parasites in mice showed no phenotype during acute infection but an altered cytokine response during long-term chronic infection and a localization change of TgPL1 to the cyst wall (44). Unlike TgPL1, the absence of TgLOXL1 results in parasite clearance during acute infection, but the mechanism is still unknown.

A decrease in *T. gondii* replication rate in macrophages stimulated with LPS and IFN-γ has previously been reported (8, 45). We noted that BMDMs stimulated with both LPS and IFN-γ contained only a single parasite per vacuole whereas naïve, LPS, or IFN-γ stimulated BMDMs contained multi-parasite vacuoles (Fig. 7A). We quantified the replication rate in stimulated BMDMs and found that all three strains had the greatest reduction in their replication rates in macrophages stimulate them with both LPS and IFN-γ (Fig. S6C and D). Similar to fibroblast cells (Fig. S1B), we noticed a trend toward a reduced replication rate of ΔTgLOXL1 parasites compared to the parental strain (Fig. S6D) in naïve and activated macrophages, but this trend was not significant or consistent between all experiments (Fig. S6D and E). To test if the concentration of IFN-γ could influence the replication pattern, we incubated infected BMDMs with increasing concentrations of IFN-γ, 100 ng/mL LPS or both 100 U/mL IFN-γ and 100 ng/mL LPS together (Fig. S6F). Our data indicated a reduced parasite replication of parental and ΔTgLOXL1 parasites as IFN-γ increased, which were not significant between strains at any concentration (Fig. S6F). To our knowledge, this is the first report of a *T. gondii* potential lipoxygenase and its importance *in vivo*. There is still much to be discovered in the association between the parasite’s lipid metabolism and its role in immune evasion.

## MATERIALS AND METHODS

### Parasite and host cell culture

*T. gondii* tachyzoites were cultured in human foreskin fibroblasts in Dulbecco’s modified Eagle’s medium (DMEM, Gibco) supplemented with 10% fetal bovine serum, 2 mM L-glutamine, and 1% penicillin-streptomycin at 37°C with 5% CO_2_. To avoid the appearance of any tissue culture specific characteristics, low passage parasites were frozen in 10% DMSO, 20% FBS, and 70% DMEM and used until passage 15.

### Sequence alignment and candidate gene selection

To select the potential LOX candidates involved in the sexual development of *T. gondii,* we performed a database search. Annotated protein data from the *T. gondii* TGME49 strain was downloaded from ToxoDB.org, and the amino acid sequence of each protein was scanned for the presence of a C-terminal isoleucine or valine. The resulting protein IDs from this scan were then used in a search strategy on ToxoDB. The gene ID was cross-referenced with transcripts that were more than 100-fold upregulated in the sexual stage compared to the tachyzoite stage using the feline enterocyte, tachyzoite, bradyzoite stage transcriptome (16). Then the candidates were organized from highest to lowest TPM value, and our candidate gene was selected.

### Generation of TgLOXL1 knockout and complement strains

A pBC_TubCAT_HXGPRT empty vector was digested with SpeI and KpnI to insert the flanking regions of our gene of interest on either side of the chloramphenicol acetyl transferase (CAT) positive selectable marker, with hypoxanthine-xanthine-guanine phosphoribosyl transferase (HXGPRT) as the negative selectable marker. The 4 kb genomic fragments 5’ and 3’ of the TgLOXL1 locus were amplified by PCR with Phusion polymerase (Thermo Scientific) using primers LOXL1 5’ flank F (actagtcgtcctccctagcaataac), LOXL1 5’ flank R (actagtggagacttgtcgtcactcttg), LOXL1 3’ flank F (ggtaccgttagacggtgatggtggtatgc), and LOXL1 3’ flank R (ggtaccctcacggaggaagattgtttg). The resulting pBC_TubCAT_HXGPRT_315970 vector was linearized prior to electroporation of parental PruΔHPT:luciferase parasites (43) with the BTX ECM 630 Electroporation System. Successful knockout strains were selected with chloramphenicol and 6-thioxanthine, and clonal populations were isolated by limiting dilution. Loss of the full TgLOXL1 gene was confirmed by PCR using primers LOXL1 KO F (ggctacggacatcggtca) and LOXL1 KO R (ggtgaggtgacatcggtaca). Complement strains were created by amplifying cDNA from the TgLOXL1 coding region using the primers LOXL1 ORF F (gaattcatggccatgcaactgccag) and LOXL1 ORF R (ttaattaaattataggtgcccttctgctccg) and inserting the coding region into a pBC_SK vector containing a tubulin promoter and SAG1 3’ UTR with a DHFR positive selectable marker. The resulting pBC_SK_DHFR_315970 vector was linearized prior to electroporation with the BTX ECM 630 Electroporation System. Successful complement strains were selected for non-homologous recombination with pyrimethamine, and clonal populations were isolated by limiting dilution. The presence of the TgLOXL1 coding region was confirmed by PCR using LOXL1 KO F/R primers.

### Plaque assay

Plaque assays were performed by seeding HFF cells in 12-well plates until a confluent monolayer was formed. Cells were infected with 500 parasites of parental PruΔHPT:luciferase, ΔTgLOXL1, or complement untagged parasite strains, and left undisturbed for 7 d for the plaques to form. The medium was removed, and the cells were fixed with methanol and stained with crystal violet for 20 min. Then the samples were rinsed with water and left to air dry overnight and then photographed. Plaques were counted using ImageJ software by analyzing the number of individual plaques formed.

### Replication assay

Replication assays were performed by seeding HFF cells on sterile glass coverslips in 24-well plates and infecting the monolayer with 10^5^ parasites of parental PruΔHPT:luciferase, ΔTgLOXL1, or complement untagged parasite strains in triplicate. After 2 h of incubation, each well was washed with PBS to remove extracellular parasites, and then new media was added. After 12, 24, or 36 h post-infection, the samples were fixed and stained with 1:500 rabbit anti-SAG1 primary antibody and 1:1,000 goat anti-rabbit Alexa Fluor 488 and counterstained with DAPI. After mounting, the slides were blinded, and the number of parasites per vacuole was counted. The statistics were performed using Analysis of Variance in the GraphPad Prism software.

### Mouse infections and parasitemia measurements

Mice from 8- to 12-wk-old were used for all the experiments, and to the best of our abilities we maintained the same proportion of male and female mice to account for sex-dependent variability. For the infections, parasites were scraped and syringed from HFF monolayers and counted for i.p. injection of parental PruΔHPT:luciferase, ΔTgLOXL1, or complement untagged parasite strains. The number of parasites used for infection varied depending on the experiment being performed and the strain of mice used (WT vs IFN-γ KO).

#### Survival curves

Between 4 and 5 mice were used for each parasite strain and the experiment was repeated at least twice. NMRI and C57BL/6 wild-type mice were inoculated via i.p. injection with 1 × 10^4^ or 1 × 10^5^ parasites. IFN-γ KO mice were inoculated with 1 × 10^2^–1×10^4^ parasites due to their higher susceptibility to infection. Mice were monitored daily up to 28 d post-infection for disease symptoms scored on a 1–4 scale, with 1 indicating no pain and distress and 4 indicating moribund condition (46). Surviving mice were checked for parasitemia. Prism GraphPad software was used to analyze survival curves by log-rank (Mantel-Cox).

#### Measuring acute parasitemia by IVIS

Between 4 and 5 mice were used for each parasite strain and the experiment was repeated at least twice. WT mice were inoculated by i.p. injection with 1 × 10^5^ or 1 × 10^6^ parasites, and IFN-γ KO mice were inoculated with 1 × 10^4^ parasites. At 7 d post-infection parasitemia in mice was detected by bioluminescence using IVIS (PerkinElmer). Wild-type mice infected with the higher parasite dose of 1 × 10^6^ parasites were imaged at 3 d post-infection. Prior to imaging, mice were injected i.p. with 200 µL of D-Luciferin potassium salt (15.4 mg/mL in PBS) and anesthetized with 4% isoflurane. Anesthesia was maintained throughout the imaging process at 2% isoflurane. After 15 min images were collected, and the mice were returned to their cages to recover. A background measurement was determined by injecting an uninfected mouse with D-Luciferin and collecting the images as previously described. Data collection was performed by drawing a region of interest (ROI) box for each mouse and recording the total number of photons per second (Total Flux) (47).

#### Chronic infection parasitemia by luciferase and DBA

Experiments were performed using NMRI and Swiss Webster mice inoculated via i.p. injection with 1 × 10^4^ parasites. After 28 d, brains were collected and homogenized in 1 mL of ice-cold PBS. For the luciferase assay, a 750 µL aliquot from the sample was transferred to a clean Eppendorf tube and mixed with 250 µL of 5 × lysis buffer (125 mM Tris, 10 mM DTT, 50% Glycerol, 5% Triton-X100) and incubated for 20 min in ice. The sample was mixed, separated into two 500 µL aliquots, and left on ice. A stock of 2 × luciferase reaction buffer (10 mM MgCl_2_, 0.3 mM ATP, 200 mM, Luciferin 1.5 mg/mL final concentration) was prepared, left at room temperature, and protected from light until samples were ready to be measured. Each sample aliquot was mixed with 500 µL of the 2 × luciferase reaction buffer, mixed by inversion, and its luminescence was measured on a GloMax 20/20 Luminometer (Promega). For the DBA assay, the samples were fixed in 3.7% formaldehyde in PBS for 20 min and permeabilized and blocked with 0.2% vol/vol Triton x-100 (Sigma) and 3% BSA in PBS at room temperature for 1 h The samples were incubated with a 1:500 dilution of biotinylated DBA (Vector Laboratories) followed by a 1:500 dilution of streptavidin Alexa Fluor 594 (Thermofisher) for 1 hour each, washing after each incubation.

#### Mice treated with Meloxicam

Between 3 and 5 C57BL/6 mice were used for each parasite strain and the experiment was repeated at least twice. WT mice were treated daily by i.p. injection with 10 mg/kg Meloxicam (Cayman Chemical Company) dissolved in 1% DMSO in PBS. A day after the first treatment, mice were either left uninfected or inoculated by i.p. injection with 1 × 10^4^ parasites. At 7 d blood serum was collected by final bleed. As control, untreated mice were also injected with the vehicle. The serum was stored at −20°C, and the cytokine levels were measured.

### Cytokine bead assay

Serum cytokine levels were measured using the BD cytometric bead array mouse inflammation kit (BD Biosciences). Blood serum was collected from the mice used in the IVIS experiment on days 0, 3, or 7 post-infection. To test TgLOXL1 during lipid metabolism as an immune regulator, blood serum was collected from mice treated with Meloxicam at 7 d post-infection and serum cytokine levels were measured. Samples were processed according to the manufacturer’s instructions and analyzed using an Attune flow cytometer (Thermo-Fisher) at the University of Wisconsin Carbone Cancer Center. Further analysis was performed using the FlowJo software. The statistics were performed using one-way ANOVA in the GraphPad Prism software.

### Western blot

HFF monolayers were infected, and after 24 h post-infection cells were scraped and lysed by a syringe (28G needle). Detached cells were then centrifuged 500 × g for 10 min, and the pellet was solubilized using lysis buffer (2% 2-mercaptoethanol, 1% SDS, 20 mM EGTA, 2 mM Tris–HCl at pH 7.5, 0.1 mM PMSF, 0.1 mM TPCK, and 0.1 mM TLCK) and sonicated in ice for 15 s at 40 Hz (Branson Sonifier 250). Approximately, 20 µg of protein were loaded into a gel (SDS-PAGE), transferred to a nitrocellulose membrane, and blocked with 6% skim milk dissolved in TBS-T (10 mM Tris–HCl, 75 mM NaCl, and 0.1% Tween 20 at pH 8.0,) for 1 h. Primary antibody against-SAG1 (rabbit, 1:3,000 kindly donated by John Boothroyd), −HA Tag (mouse monoclonal, Enzo Life Sciences, NY) was incubated overnight at 4°C and then thoroughly washed with TBS-T. Secondary antibodies conjugated to horseradish peroxidase (HRP, rabbit or mouse 1:7,000, Thermo Scientific) were incubated for 2 h at room temperature. Antibody 6xHisTag was already conjugated to HRP (Goat polyclonal, 1:2,000, Bethyl Laboratories). Chemiluminescence (ECL. GE, Buckingham-shire, UK) was detected using the Odyssey XK Instrument (LI-COR Imaging system).

### Indirect immunofluorescence assays

To obtain the samples: (i) HFF monolayers or BMDMs were seeded on coverslips in 24 well-plates and infected as described above, (ii) C57BL/6 mice were infected via i.p. injection with 2 × 10^6^ parasites, and after 3 d, and infected leukocytes were isolated by peritoneal lavage, or (iii) extracellular parasites were obtained by infection of T25 flasks with confluent HFF monolayers with 1 × 10^6^ N-terminal HA-tagged parasites; parasites were collected until they naturally egress (3 dpi). Cells were fixed with 4% paraformaldehyde (PFA), permeabilized with 0.5% Triton X-100, and blocked with 5% BSA. To test the integrity of PVM, cells were treated with saponin 0.005% for 20 min at 4°C which only permeabilizes the host plasma membrane (20). Cells were incubated with the primary antibodies against- SAG1 (rabbit, 1:300), -MIC2 (rabbit, 1:300), -GRA2 (rabbit, 1:500), -GRA3 (rabbit, 1:500), -GRA4 (rabbit, 1:500), -luciferase (mouse monoclonal, 1:1,000, Sigma), or HA-Tag (mouse monoclonal, 1:1,000) overnight at 4 ˚C and then thoroughly washed with PBS. Secondary antibodies against -Alexa Fluor 488 (Rabbit anti-mouse, 1:1,000, Thermo Scientific) and -Alexa Fluor 594 (Goat anti-rabbit, 1:1,000, Thermo Scientific) were incubated for 2 h at room temperature. Leukocytes were seeded on poly-L-lysine coated coverslips. Cells were counterstained with DAPI and mounted using VECTASHIELD antifade Mounting Medium (Vector Laboratories Inc., CA). Cells were imaged using an epifluorescence microscope (Imager.M2, Carl Zeiss, DEU) or a confocal microscope (ZeissLSM 800 Laser Scanning Microscope). 3D projections were reconstructed from z-stack sections using the Zen imaging software (Carl Zeiss).

### Quantification of TgLOXL1-HA in stimulated BMDMs

BMDMs were harvested from 8- to 10-week-old mice and grown in 20% L929 conditioned RPMI medium as described previously (48). A total of 1 × 10^5^ viable BMDMs were seeded on poly-L-lysine coated coverslips and infected with 1  ×  10^5^ parental PruΔHPT:luciferase, ΔTgLOXL1, complement untagged, and N-terminal HA-tag tachyzoites. After 3  h post-infection, cells were either left naive or stimulated with 20–100U/mL IFN-γ or 100 U/mL IFN-γ, and 100  ng/mL LPS or 100 ng/mL LPS. At 48  h post-infection, cells were fixed, processed, and imaged as above described. Total quantification of HA in BMDMs was performed by measuring the total fluorescence intensity of HA (green channel) and normalized against total fluorescence intensity of SAG1 (red channel). The background of the green and red in the naïve sample was subtracted from the data of stimulated BMDMs. To quantify the amount of TgLOXL1-HA released by the parasite, intra-parasite TgLOXL1-HA and total TgLOXL1-HA (parasite and host) were quantified, and the ratio was determined. As a control, we considered a subset of the wells with BMDMs uninfected and/or unstimulated, which were processed and analyzed equally. Quantification of fluorescence was performed in ImageJ. Data were graphed in the GraphPad Prism software and is shown in arbitrary units. The statistics were performed using one-way ANOVA in the GraphPad Prism software.

### Quantification of *T. gondii* proliferation in BMDMs

To test the parasite proliferation under classical activation of BMDMs, 1 × 10^5^ BMDMs were seeded in a 24-well plate and allowed to settle and attach for 30 min. Then, BMDMs were infected with 1 × 10^5^ parasites of parental, ΔTgLOXL1, and complement strains. At 3 h post-infection, the medium was changed to removed extracellular parasites. BMDMs were activated with 100 ng/mL lipopolysaccharide and 100 U/mL IFN-γ independently and together to mimic classical activation. After 48 h post-infection, BMDMs were fixed, permeabilized, blocked, and incubated with SAG1 antibody as mentioned above. BMDMs were subsequentially counterstained with SYTOX Green Nucleic Acid Stain (Thermo Scientific), in the dark at room temperature for 20 min. Cells were again rinsed and placed for imaging on the IncuCyte analyzer. For all replicates and samples, the red channel used an acquisition time of 300 ms (ms), and the green channel was set to 250 ms to minimize background fluorescence. The host nuclei (green) were analyzed with custom IncuCyte parameters using a threshold of 2.10 Green Calculated Units (GCUs) with adaptive segmentation, and the parasites (red) were measured at a threshold of 0.90 Red Calculated Units (RCUs) with surface-fit segmentation. All the conditions were analyzed in duplicate. As a control, we considered a subset of the wells with BMDMs uninfected and/or unstimulated, which were processed and analyzed equally. Statistics were performed using one-way ANOVA and were graphed in the GraphPad Prism software.

To test the parasite proliferation under different concentrations of IFN-γ, parasite growth was determined by immunofluorescence in BMDMs. A total of 1 × 10^5^ viable BMDMs were seeded on poly-L-lysine coated coverslips and infected with 1  ×  10^5^ parental PruΔHPT:luciferase, ΔTgLOXL1, and complement untagged parasites. After 3  h post-infection, cells were either left naive or stimulated with 20–100U/mL IFN-γ or 100 U/mL IFN-γ, and 100  ng/mL LPS or 100 ng/mL LPS. At 48  h post-infection, cells were fixed and processed for staining with *T. gondii* SAG1 as described above and imaged for quantification of parasite per vacuole. Each condition was done in duplicate and by photographing five random fields per coverslip with a Zeiss Axioplan III motorized microscope with a 40 × objective. All photographs were blinded before counting. All parasites per vacuole were counted for every vacuole in the photographs. Statistics were performed using one-way ANOVA and were graphed in the GraphPad Prism software.

## SUPPLEMENTAL MATERIAL

Fig. S1 and S2 mbio.01279-23-s0001.pdf Generation of the knockout strain ΔTgLOXL1, its growth in tissue culture, and localization in the cytoplasm of intracellular tachyzoites.

Fig. S3 mbio.01279-23-s0002.pdf ΔTgLOXL1 parasites have reduced parasitemia during chronic infection.

Fig. S4 mbio.01279-23-s0003.pdf ΔTgLOXL1 parasitemia is reduced even with large inoculums.

Fig. S5 mbio.01279-23-s0004.pdf MCP-1 and IL-6 are upregulated in the serum of IFN-γ KO mice during acute infection.

Fig. S6 mbio.01279-23-s0005.pdf Intracellular proliferation of *T. gondii* in BMDMs.

All Legends mbio.01279-23-s0006.docx Legends for the six supplemental figures and three supplemental movies.

Movie S1 mbio.01279-23-s0007.mp4 TgLOX1 localized in the cytoplasm of intracellular tachyzoites. 3D projections of parasites expressing HA-tag in the N-terminus TgLOX1 (green) and the parasite surface localization of SAG1 (red) in intracellular tachyzoites at 24 hours postinfection and visualized by confocal microscopy.

Movie S2 mbio.01279-23-s0008.mp4 TgLOX1 is released by the parasite inside infected leukocytes. 3D projections of infected leukocytes isolated from the peritoneal cavity of infected mice. Mice were infected with 2x106 N-terminal HA-tag parasites and 3 days postinfection leukocytes were isolated. Parasites were stained for SAG1 (red) and TgLOX1-HA (green), and nuclei were counterstained with DAPI.

Movie S3 mbio.01279-23-s0009.mp4 Parasite inside infected leukocytes. 3D projections of infected leukocytes isolated from the peritoneal cavity of infected mice. Mice were infected with 2x106 N-terminal HA-tag parasites and 3 days postinfection leukocytes were isolated. Parasites were stained for SAG1 (red) and TgLOX1-HA (green), and nuclei were counterstained with DAPI.

ASM does not own the copyrights to Supplemental Material that may be linked to, or accessed through, an article. The authors have granted ASM a non-exclusive, world-wide license to publish the Supplemental Material files. Please contact the corresponding author directly for reuse.

## References

[1] Flegr J , Prandota J , Sovičková M , Israili ZH . 2014. Toxoplasmosis-a global threat. correlation of latent toxoplasmosis with specific disease burden in a set of 88 countries. PLoS One 9:e90203. doi:10.1371/journal.pone.0090203 24662942 PMC3963851

[2] Mennechet FJD , Kasper LH , Rachinel N , Li W , Vandewalle A , Buzoni-Gatel D . 2002. Lamina propria CD4^+^ T lymphocytes synergize with murine intestinal epithelial cells to enhance proinflammatory response against an intracellular pathogen. J Immunol 168:2988–2996. doi:10.4049/jimmunol.168.6.2988 11884471

[3] Gazzinelli RT , Wysocka M , Hayashi S , Denkers EY , Hieny S , Caspar P , Trinchieri G , Sher A . 1994. Parasite-induced IL-12 stimulates early IFN-gamma synthesis and resistance during acute infection with Toxoplasma gondii. J Immunol 153:2533–2543. doi:10.4049/jimmunol.153.6.2533 7915739

[4] Suzuki Y , Orellana MA , Schreiber RD , Remington JS . 1988. Interferon-gamma: the major mediator of resistance against Toxoplasma gondii. Science 240:516–518. doi:10.1126/science.3128869 3128869

[5] Gilroy DW , Bishop-Bailey D . 2019. Lipid mediators in immune regulation and resolution. Br J Pharmacol 176:1009–1023. doi:10.1111/bph.14587 30674066 PMC6451072

[6] Nozaki M , Ishimura Y . 1974. Oxygenases, p 417–444. In Neilands JB (ed), Microbial iron metabolism. Elsevier.

[7] Norman AW , Henry HL . 2015. Eicosanoids, p 171–188. In Norman AW , HL Henry (ed), Hormones, 3rd ed. Elsevier.

[8] Pereira ACA , Silva RJ , Franco PS , de Oliveira Gomes A , Souza G , Milian ICB , Ribeiro M , Rosini AM , Guirelli PM , Ramos ELP , Mineo TWP , Mineo JR , Silva NM , Ferro EAV , Barbosa BF . 2019. Cyclooxygenase (COX)-2 inhibitors reduce Toxoplasma gondii infection and upregulate the pro-inflammatory immune response in calomys callosus rodents and human monocyte cell line. Front Microbiol 10:225. doi:10.3389/fmicb.2019.00225 30809216 PMC6379304

[9] de Souza G , Silva RJ , Milián ICB , Rosini AM , de Araújo TE , Teixeira SC , Oliveira MC , Franco PS , da Silva CV , Mineo JR , Silva NM , Ferro EAV , Barbosa BF . 2021. Cyclooxygenase (COX)-2 modulates Toxoplasma gondii infection, immune response and lipid droplets formation in human trophoblast cells and villous explants. Sci Rep 11:12709. doi:10.1038/s41598-021-92120-3 34135407 PMC8209052

[10] Aliberti J , Hieny S , Reis e Sousa C , Serhan CN , Sher A . 2002. Lipoxin-mediated inhibition of IL-12 production by DCs: a mechanism for regulation of microbial immunity. Nat Immunol 3:76–82. doi:10.1038/ni745 11743584

[11] Aliberti J , Serhan C , Sher A . 2002. Parasite-induced lipoxin A4 is an endogenous regulator of IL-12 production and immunopathology in Toxoplasma gondii infection. J Exp Med 196:1253–1262. doi:10.1084/jem.20021183 12417634 PMC2194099

[12] Horowitz Brown S , Zarnowski R , Sharpee WC , Keller NP . 2008. Morphological transitions governed by density dependence and lipoxygenase activity in Aspergillus flavus. Appl Environ Microbiol 74:5674–5685. doi:10.1128/AEM.00565-08 18658287 PMC2547031

[13] Di Genova BM , Wilson SK , Dubey JP , Knoll LJ . 2019. Intestinal delta-6-desaturase activity determines host range for Toxoplasma sexual reproduction. PLos Biol. doi:10.1101/688580 PMC6701743 31430281

[14] Minor W , Steczko J , Stec B , Otwinowski Z , Bolin JT , Walter R , Axelrod B . 1996. Crystal structure of soybean lipoxygenase L-1 at 1.4 a resolution. Biochem 35:10687–10701. doi:10.1021/bi960576u 8718858

[15] Newcomer ME , Brash AR . 2015. The structural basis for specificity in lipoxygenase catalysis. Protein Sci 24:298–309. doi:10.1002/pro.2626 25524168 PMC4353356

[16] Ramakrishnan C , Maier S , Walker RA , Rehrauer H , Joekel DE , Winiger RR , Basso WU , Grigg ME , Hehl AB , Deplazes P , Smith NC . 2019. An experimental genetically attenuated live vaccine to prevent transmission of Toxoplasma gondii by cats. Sci Rep 9:1474. doi:10.1038/s41598-018-37671-8 30728393 PMC6365665

[17] Chen XS , Kurre U , Jenkins NA , Copeland NG , Funk CD . 1994. cDNA cloning, expression, mutagenesis of C-terminal isoleucine, genomic structure, and chromosomal localizations of murine 12-lipoxygenases. J Biol Chem 269:13979–13987. doi:10.1016/S0021-9258(17)36743-1 8188678

[18] Mordue DG , Scott-Weathers CF , Tobin CM , Knoll LJ . 2007. A patatin-like protein protects Toxoplasma gondii from degradation in activated macrophages. Mol Microbiol 63:482–496. doi:10.1111/j.1365-2958.2006.05538.x 17166175 PMC3392091

[19] Sorgi CA , Zarini S , Martin SA , Sanchez RL , Scandiuzzi RF , Gijón MA , Guijas C , Flamand N , Murphy RC , Faccioli LH . 2017. Dormant 5-lipoxygenase in inflammatory macrophages is triggered by exogenous arachidonic acid. Sci Rep 7:10981. doi:10.1038/s41598-017-11496-3 28887514 PMC5591212

[20] Black MW , Arrizabalaga G , Boothroyd JC . 2000. Ionophore-resistant mutants of Toxoplasma gondii reveal host cell permeabilization as an early event in egress. Mol Cell Biol 20:9399–9408. doi:10.1128/MCB.20.24.9399-9408.2000 11094090 PMC102196

[21] Carruthers VB , Sibley LD . 1997. Sequential protein secretion from three distinct organelles of Toxoplasma gondii accompanies invasion of human fibroblasts. Eur J Cell Biol 73:114–123. 9208224 PMC13475723

[22] Rastogi S , Cygan AM , Boothroyd JC . 2019. Translocation of effector proteins into host cells by Toxoplasma gondii. Curr Opin Microbiol 52:130–138. doi:10.1016/j.mib.2019.07.002 31446366 PMC6911000

[23] Jones TC , Alkan S , Erb P . 1986. Spleen and lymph node cell populations, in vitro cell proliferation and interferon-gamma production during the primary immune response to Toxoplasma gondii. Parasite Immunol 8:619–629. doi:10.1111/j.1365-3024.1986.tb00875.x 3101032

[24] Saeij JPJ , Coller S , Boyle JP , Jerome ME , White MW , Boothroyd JC . 2007. Toxoplasma co-opts host gene expression by injection of a polymorphic kinase homologue. Nature 445:324–327. doi:10.1038/nature05395 17183270 PMC2637441

[25] Rosowski EE , Nguyen QP , Camejo A , Spooner E , Saeij JPJ . 2014. Toxoplasma gondii inhibits gamma interferon (IFN-γ)- and IFN-β-induced host cell STAT1 transcriptional activity by increasing the association of STAT1 with DNA. Infect Immun 82:706–719. doi:10.1128/IAI.01291-13 24478085 PMC3911376

[26] Nathan C , Shiloh MU . 2000. Reactive oxygen and nitrogen intermediates in the relationship between mammalian hosts and microbial pathogens. Proc Natl Acad Sci U S A 97:8841–8848. doi:10.1073/pnas.97.16.8841 10922044 PMC34021

[27] MacMicking JD . 2012. Interferon-inducible effector mechanisms in cell-autonomous immunity. Nat Rev Immunol 12:367–382. doi:10.1038/nri3210 22531325 PMC4150610

[28] Pfefferkorn ER . 1984. Interferon gamma blocks the growth of Toxoplasma gondii in human fibroblasts by inducing the host cells to degrade tryptophan. Proc Natl Acad Sci U S A 81:908–912. doi:10.1073/pnas.81.3.908 6422465 PMC344948

[29] Pfefferkorn ER , Eckel M , Rebhun S . 1986. Interferon-gamma suppresses the growth of Toxoplasma gondii in human fibroblasts through starvation for tryptophan. Mol Biochem Parasitol 20:215–224. doi:10.1016/0166-6851(86)90101-5 3093859

[30] Dai W , Pan H , Kwok O , Dubey JP . 1994. Human indoleamine 2,3-dioxygenase inhibits Toxoplasma gondii growth in fibroblast cells. J Interferon Res 14:313–317. doi:10.1089/jir.1994.14.313 7897249

[31] Däubener W , Spors B , Hucke C , Adam R , Stins M , Kim KS , Schroten H . 2001. Restriction of Toxoplasma gondii growth in human brain microvascular endothelial cells by activation of indoleamine 2,3-dioxygenase. Infect Immun 69:6527–6531. doi:10.1128/IAI.69.10.6527-6531.2001 11553600 PMC98791

[32] Howard JC , Hunn JP , Steinfeldt T . 2011. The IRG protein-based resistance mechanism in mice and its relation to virulence in Toxoplasma gondii. Curr Opin Microbiol 14:414–421. doi:10.1016/j.mib.2011.07.002 21783405

[33] Reese ML , Shah N , Boothroyd JC . 2014. The Toxoplasma pseudokinase ROP5 is an allosteric inhibitor of the immunity-related GTPases. J Biol Chem 289:27849–27858. doi:10.1074/jbc.M114.567057 25118287 PMC4183819

[34] Fentress SJ , Behnke MS , Dunay IR , Mashayekhi M , Rommereim LM , Fox BA , Bzik DJ , Taylor GA , Turk BE , Lichti CF , Townsend RR , Qiu W , Hui R , Beatty WL , Sibley LD . 2010. Phosphorylation of immunity-related GTPases by a Toxoplasma gondii-secreted kinase promotes macrophage survival and virulence. Cell Host Microbe 8:484–495. doi:10.1016/j.chom.2010.11.005 21147463 PMC3013631

[35] Olias P , Etheridge RD , Zhang Y , Holtzman MJ , Sibley LD . 2016. Toxoplasma effector recruits the Mi-2/NuRD complex to repress STAT1 transcription and block IFN-γ-dependent gene expression. Cell Host Microbe 20:72–82. doi:10.1016/j.chom.2016.06.006 27414498 PMC4947229

[36] Gay G , Braun L , Brenier-Pinchart M-P , Vollaire J , Josserand V , Bertini R-L , Varesano A , Touquet B , De Bock P-J , Coute Y , Tardieux I , Bougdour A , Hakimi M-A . 2016. Toxoplasma gondii tgist co-opts host chromatin repressors dampening STAT1-dependent gene regulation and IFN-γ-mediated host defenses. J Exp Med 213:1779–1798. doi:10.1084/jem.20160340 27503074 PMC4995087

[37] Rommereim LM , Bellini V , Fox BA , Pètre G , Rak C , Touquet B , Aldebert D , Dubremetz J-F , Cesbron-Delauw M-F , Mercier C , Bzik DJ . 2016. Phenotypes associated with knockouts of eight dense granule gene loci (GRA2-9) in virulent Toxoplasma gondii. PLoS One 11:e0159306. doi:10.1371/journal.pone.0159306 27458822 PMC4961421

[38] Fox BA , Rommereim LM , Guevara RB , Falla A , Hortua Triana MA , Sun Y , Bzik DJ . 2016. The Toxoplasma gondii rhoptry kinome is essential for chronic infection. mBio 7:e00193-16. doi:10.1128/mBio.00193-16 27165797 PMC4959664

[39] Bai M-J , Wang J-L , Elsheikha HM , Liang Q-L , Chen K , Nie L-B , Zhu X-Q . 2018. Functional characterization of dense granule proteins in Toxoplasma gondii RH strain using CRISPR-Cas9 system. Front Cell Infect Microbiol 8:300. doi:10.3389/fcimb.2018.00300 30211128 PMC6121064

[40] Peixoto L , Chen F , Harb OS , Davis PH , Beiting DP , Brownback CS , Ouloguem D , Roos DS . 2010. Integrative genomic approaches highlight a family of parasite-specific kinases that regulate host responses. Cell Host Microbe 8:208–218. doi:10.1016/j.chom.2010.07.004 20709297 PMC2963626

[41] Scharton-Kersten TM , Wynn TA , Denkers EY , Bala S , Grunvald E , Hieny S , Gazzinelli RT , Sher A . 1996. In the absence of endogenous IFN-gamma, mice develop unimpaired IL-12 responses to Toxoplasma gondii while failing to control acute infection. J Immunol 157:4045–4054. 8892638

[42] Yap GS , Sher A . 1999. Effector cells of both nonhemopoietic and hemopoietic origin are required for interferon (IFN)-gamma- and tumor necrosis factor (TNF)-alpha-dependent host resistance to the intracellular pathogen, Toxoplasma gondii. J Exp Med 189:1083–1092. doi:10.1084/jem.189.7.1083 10190899 PMC2192999

[43] Tobin CM , Knoll LJ . 2012. A patatin-like protein protects Toxoplasma gondii from degradation in a nitric oxide-dependent manner. Infect Immun 80:55–61. doi:10.1128/IAI.05543-11 22006568 PMC3255658

[44] Tobin Magle C , Pittman KJ , Moser LA , Boldon KM , Knoll LJ . 2014. A Toxoplasma patatin-like protein changes localization and alters the cytokine response during toxoplasmic encephalitis. Infect Immun 82:618–625. doi:10.1128/IAI.00444-13 24478077 PMC3911373

[45] de Souza G , Silva RJ , Milián ICB , Rosini AM , de Araújo TE , Teixeira SC , Oliveira MC , Franco PS , da Silva CV , Mineo JR , Silva NM , Ferro EAV , Barbosa BF . 2021. Cyclooxygenase (COX)-2 modulates Toxoplasma gondii infection, immune response and lipid droplets formation in human trophoblast cells and villous explants. Sci Rep 11:12709. doi:10.1038/s41598-021-92120-3 34135407 PMC8209052

[46] Burkholder T , Foltz C , Karlsson E , Linton CG , Smith JM . 2012. Health evaluation of experimental laboratory mice. Curr Protoc Mouse Biol 2:145–165. doi:10.1002/9780470942390.mo110217 22822473 PMC3399545

[47] Saeij JPJ , Boyle JP , Grigg ME , Arrizabalaga G , Boothroyd JC . 2005. Bioluminescence imaging of Toxoplasma gondii infection in living mice reveals dramatic differences between strains. Infect Immun 73:695–702. doi:10.1128/IAI.73.2.695-702.2005 15664907 PMC547072

[48] Tobin C , Pollard A , Knoll L . 2010. Toxoplasma Gondii cyst wall formation in activated bone marrow-derived macrophages and bradyzoite conditions. J Vis Exp 42:2091. doi:10.3791/2091 PMC3156017 20736916

