# Supplementary figures and images for: A Toxoplasma gondii lipoxygenase-like enzyme is necessary for virulence and changes localization associated with the host immune response
Source: mBio. 2023 Aug 30;14(5):e01279-23. doi: 10.1128/mbio.01279-23 (PMC10653942; doi:10.1128/mbio.01279-23)

**A.**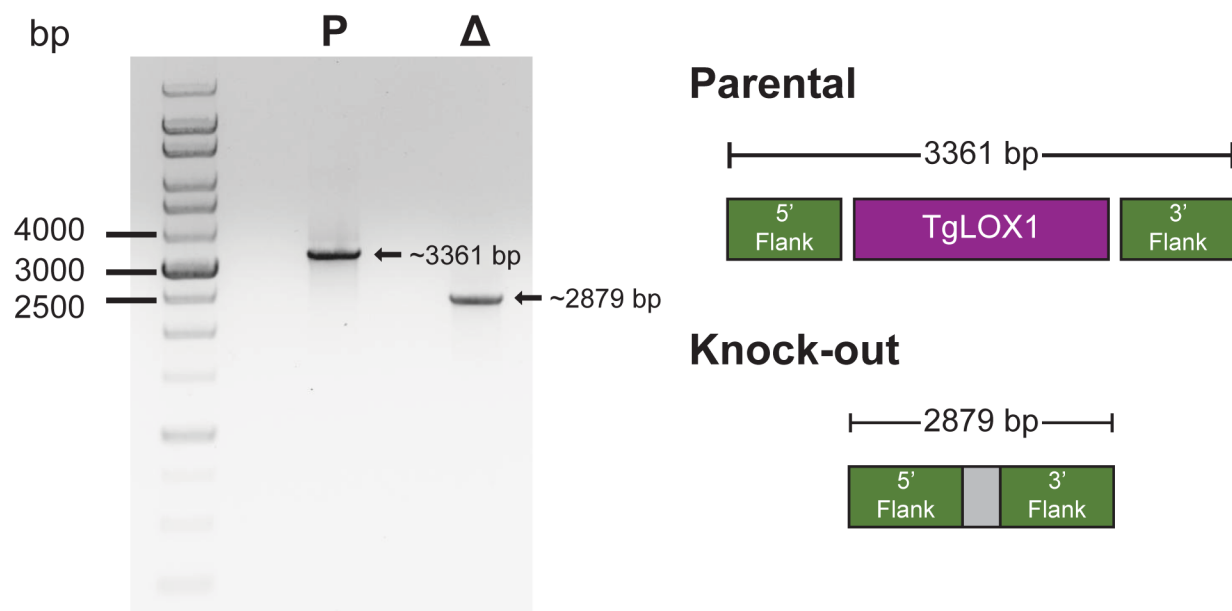**B.**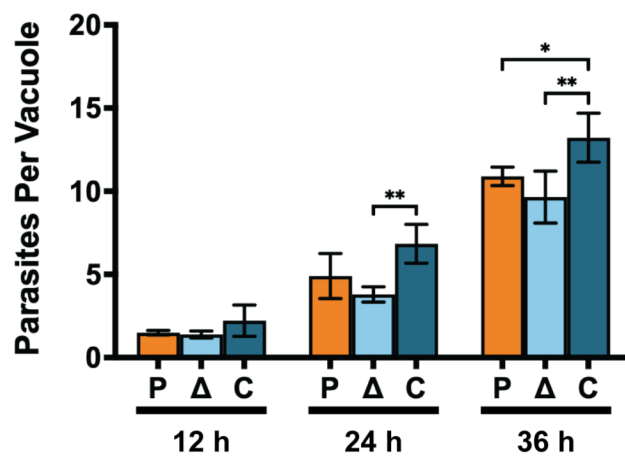**C.**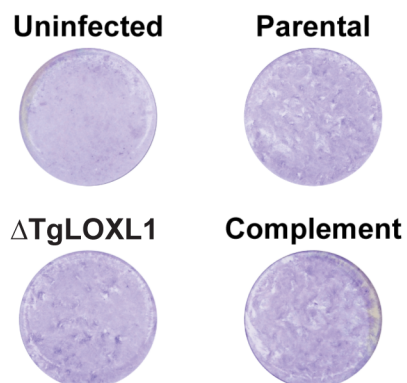**D.**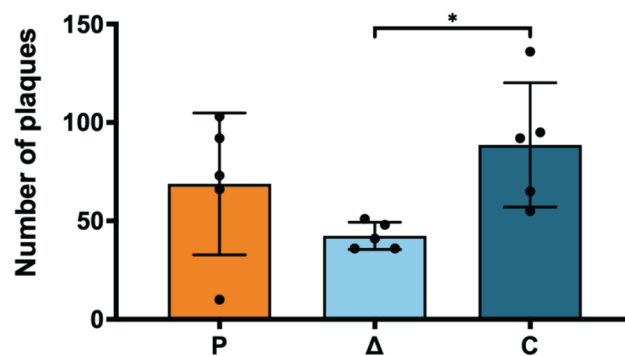

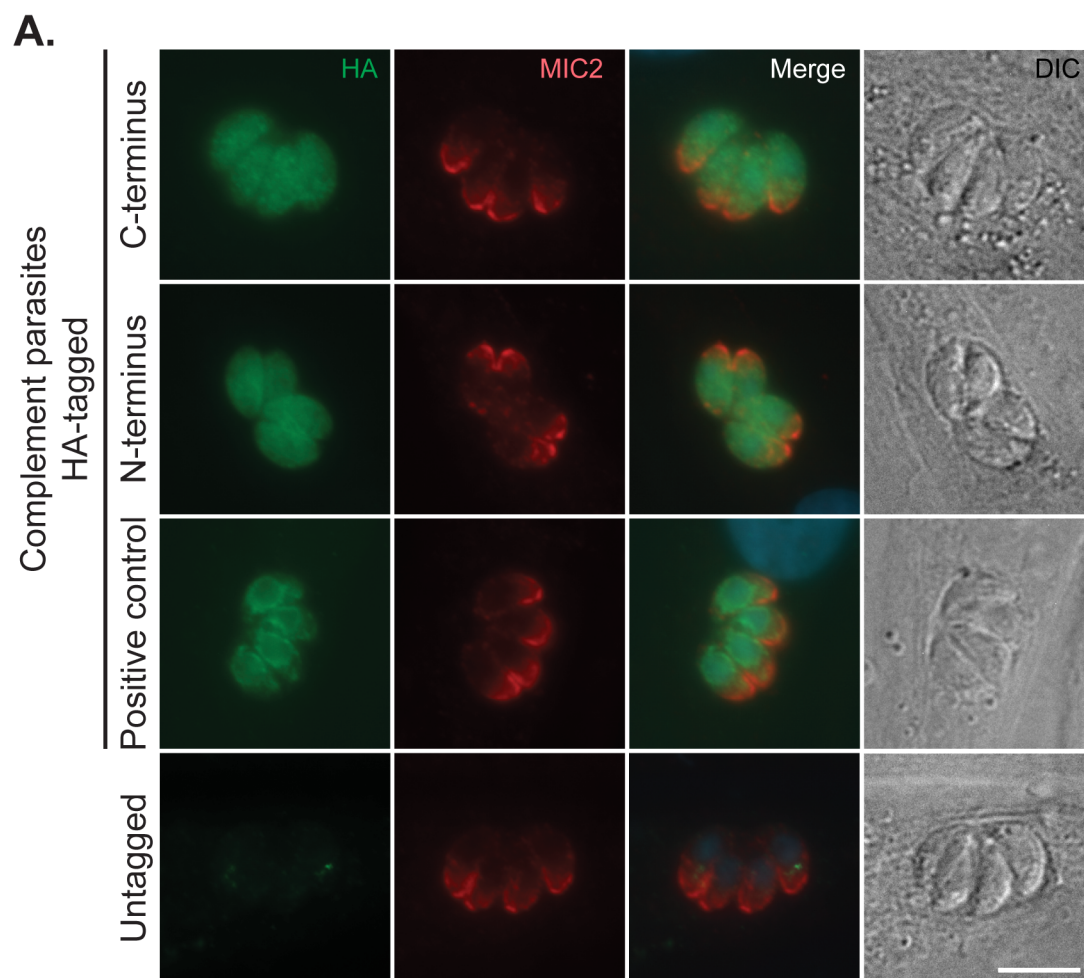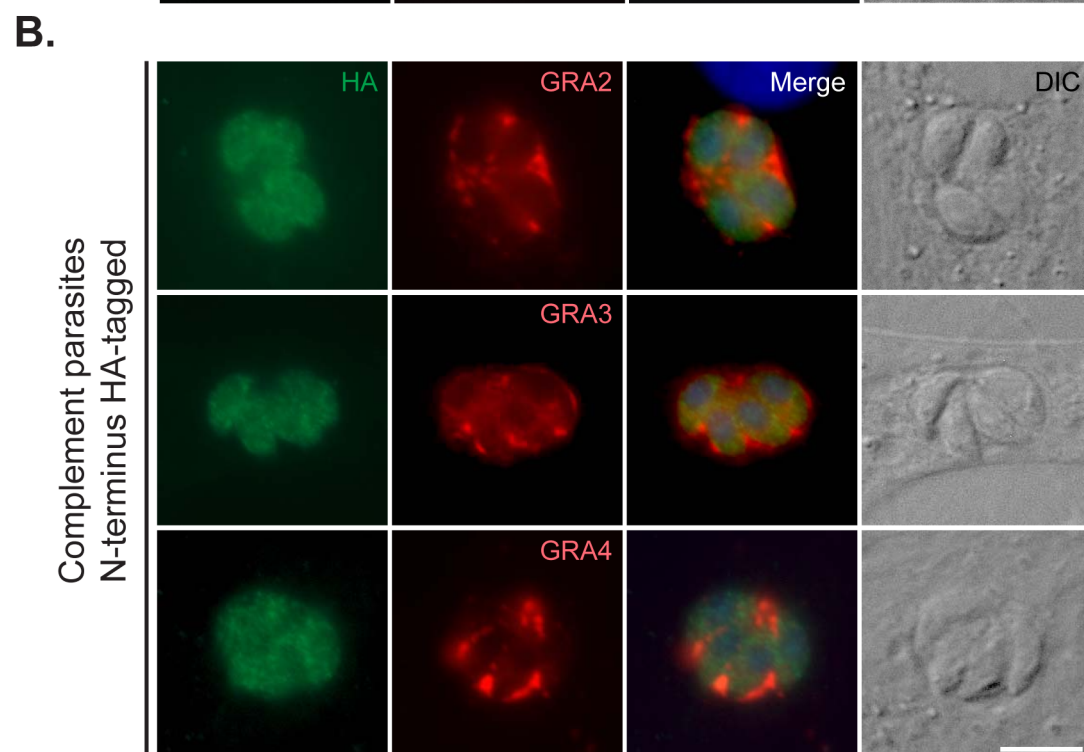

Supplement: Fig. S1 and S2 — Generation of the knockout strain ΔTgLOXL1, its growth in tissue culture, and localization in the cytoplasm of intracellular tachyzoites. [file mbio.01279-23-s0001.pdf]

## NMRI mice

A.

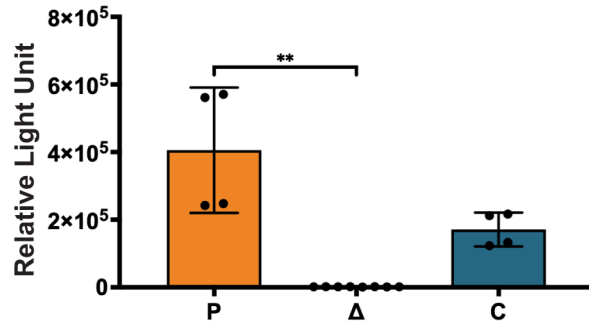

B.

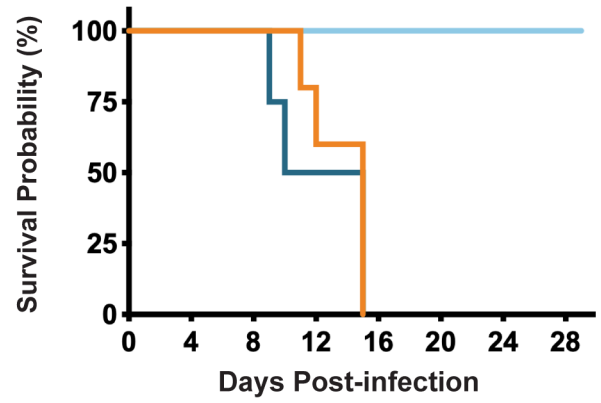

C.

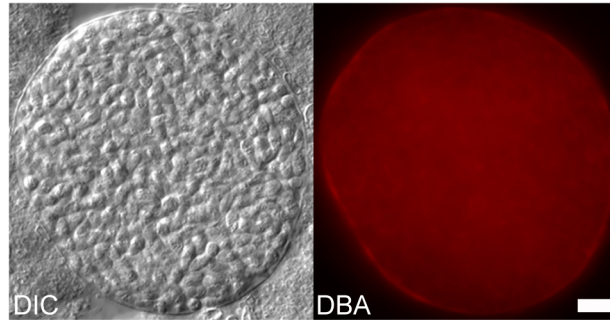

D.

## Swiss Webster mice

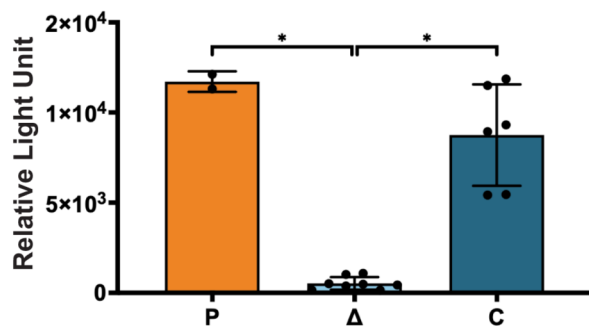

E.

## C57BL/6 wild-type mice

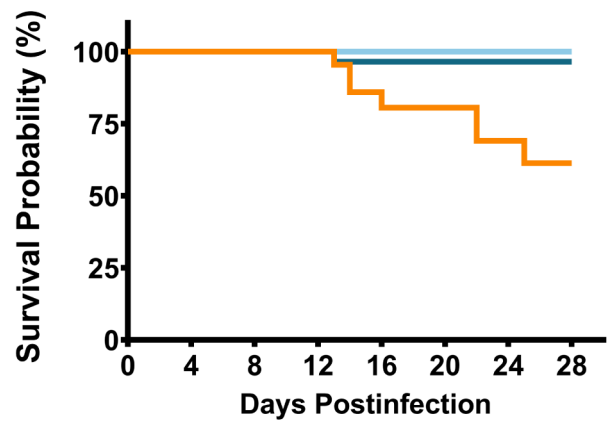

■ Parental 
 ■  $\Delta$ TgLOXL1 
 ■ Complement

Supplement: Fig. S3 — ΔTgLOXL1 parasites have reduced parasitemia during chronic infection. [file mbio.01279-23-s0002.pdf]

## C57BL/6 wild-type mice

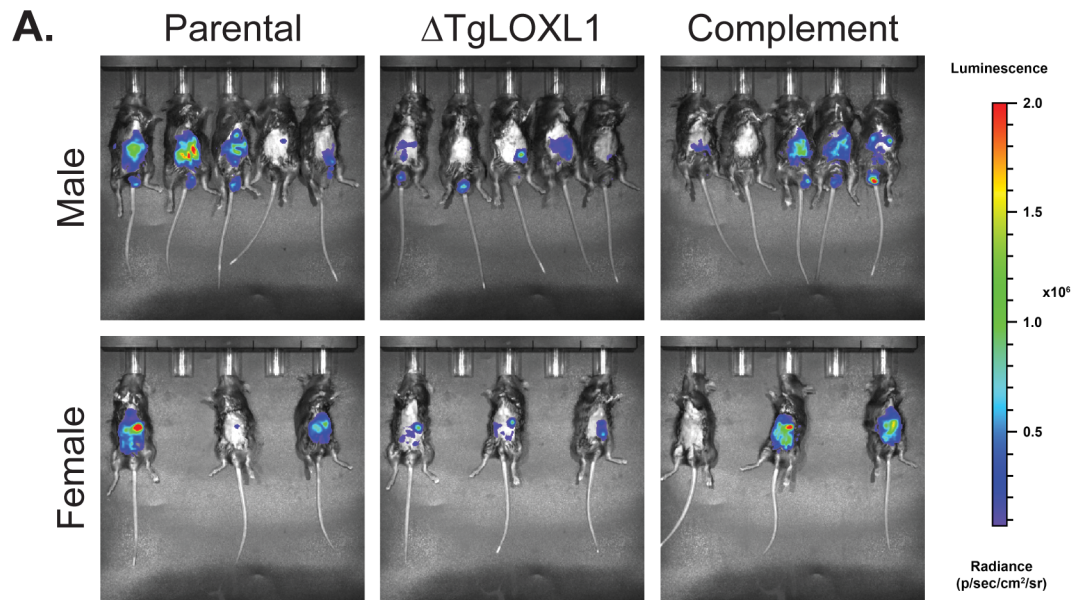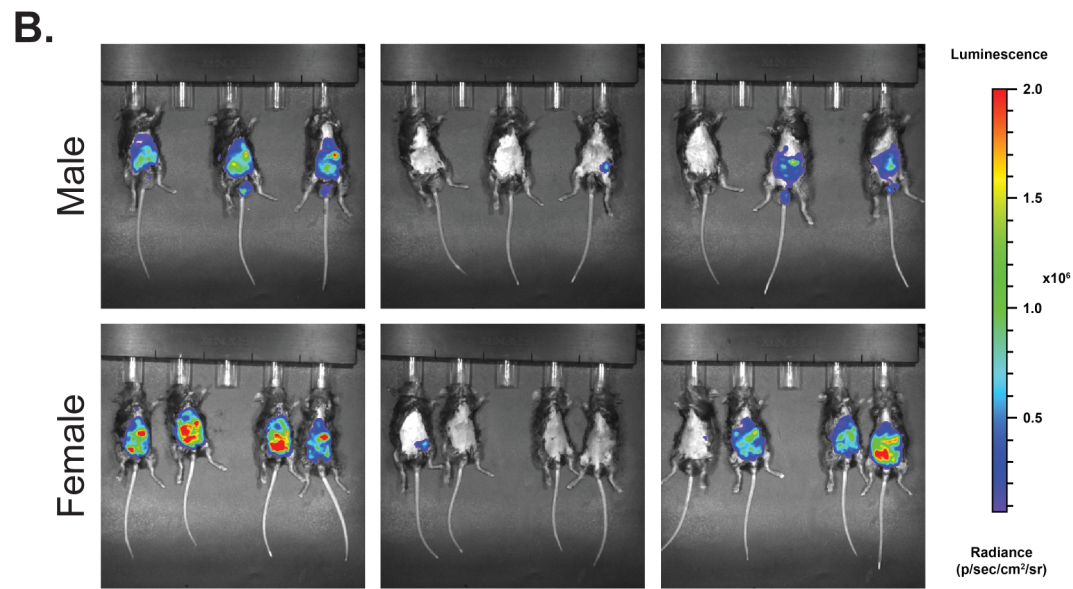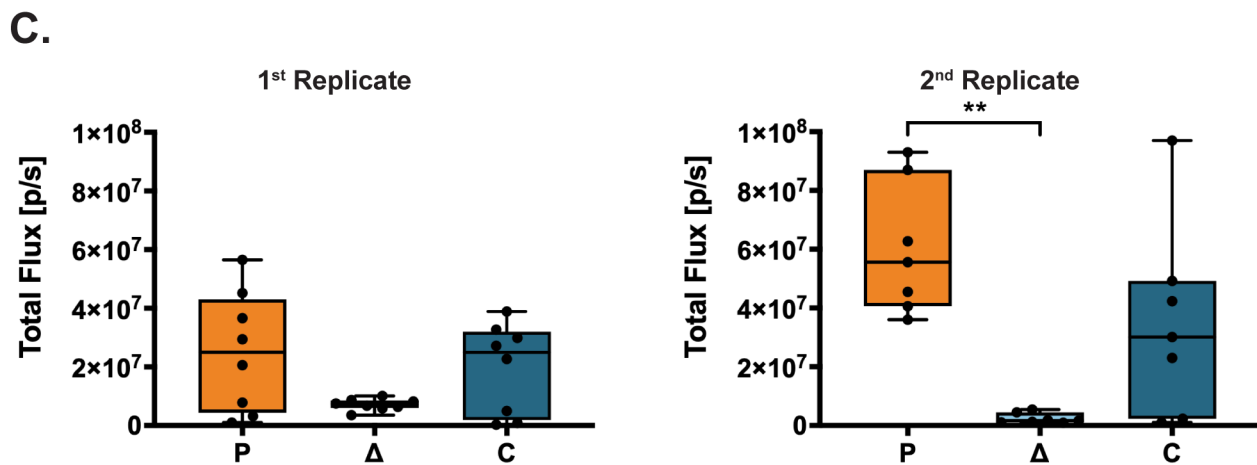

Supplement: Fig. S4 — ΔTgLOXL1 parasitemia is reduced even with large inoculums. [file mbio.01279-23-s0003.pdf]

Figure S6

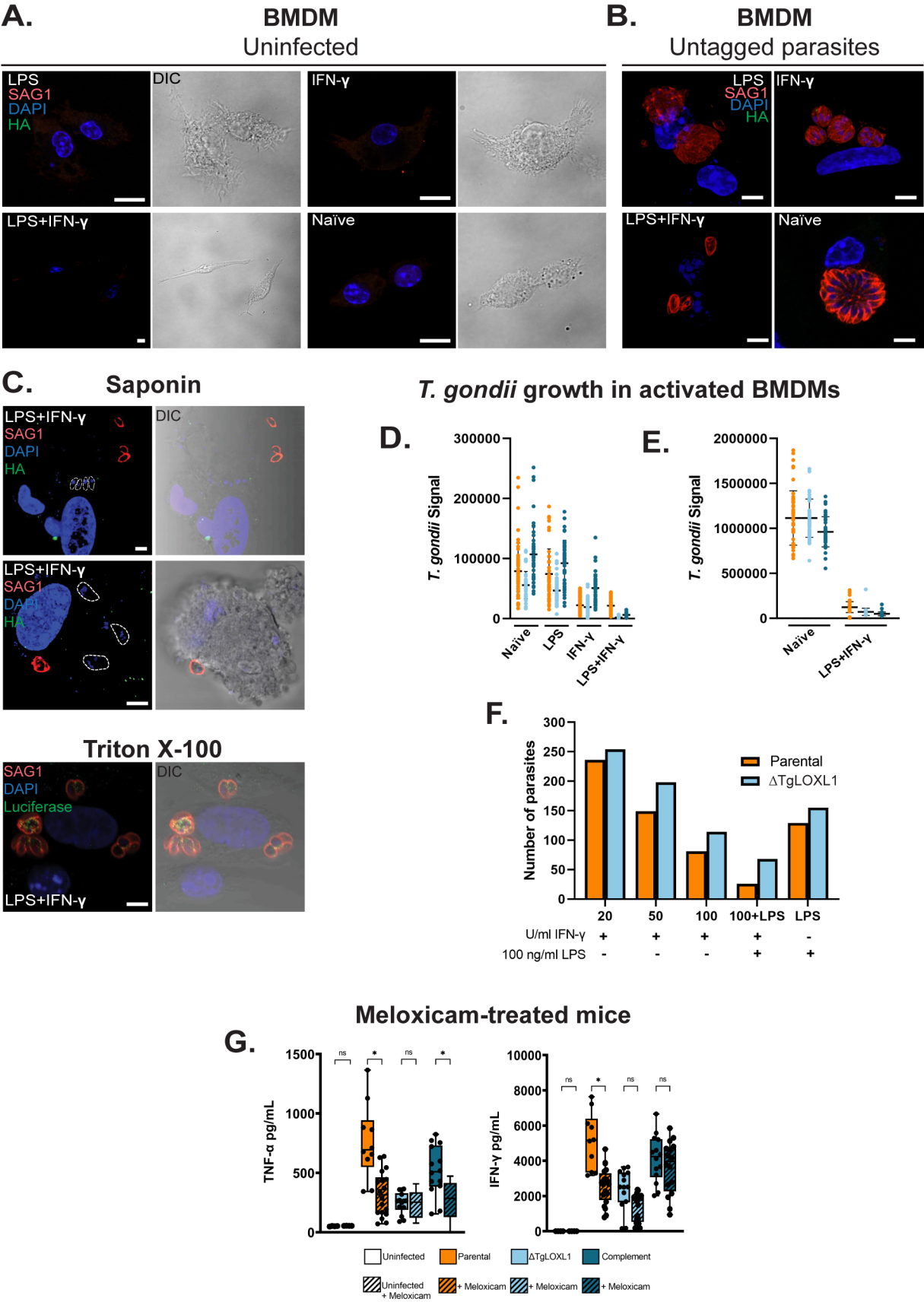

Supplement: Fig. S6 — Intracellular proliferation of T. gondii in BMDMs. [file mbio.01279-23-s0005.pdf]
