# Supplementary material for: A Toxoplasma gondii lipoxygenase-like enzyme is necessary for virulence and changes localization associated with the host immune response
Source: mBio. 2023 Aug 30;14(5):e01279-23. doi: 10.1128/mbio.01279-23 (PMC10653942; doi:10.1128/mbio.01279-23)
Supplement: Fig. S5 — MCP-1 and IL-6 are upregulated in the serum of IFN-γ KO mice during acute infection. [file mbio.01279-23-s0004.pdf]

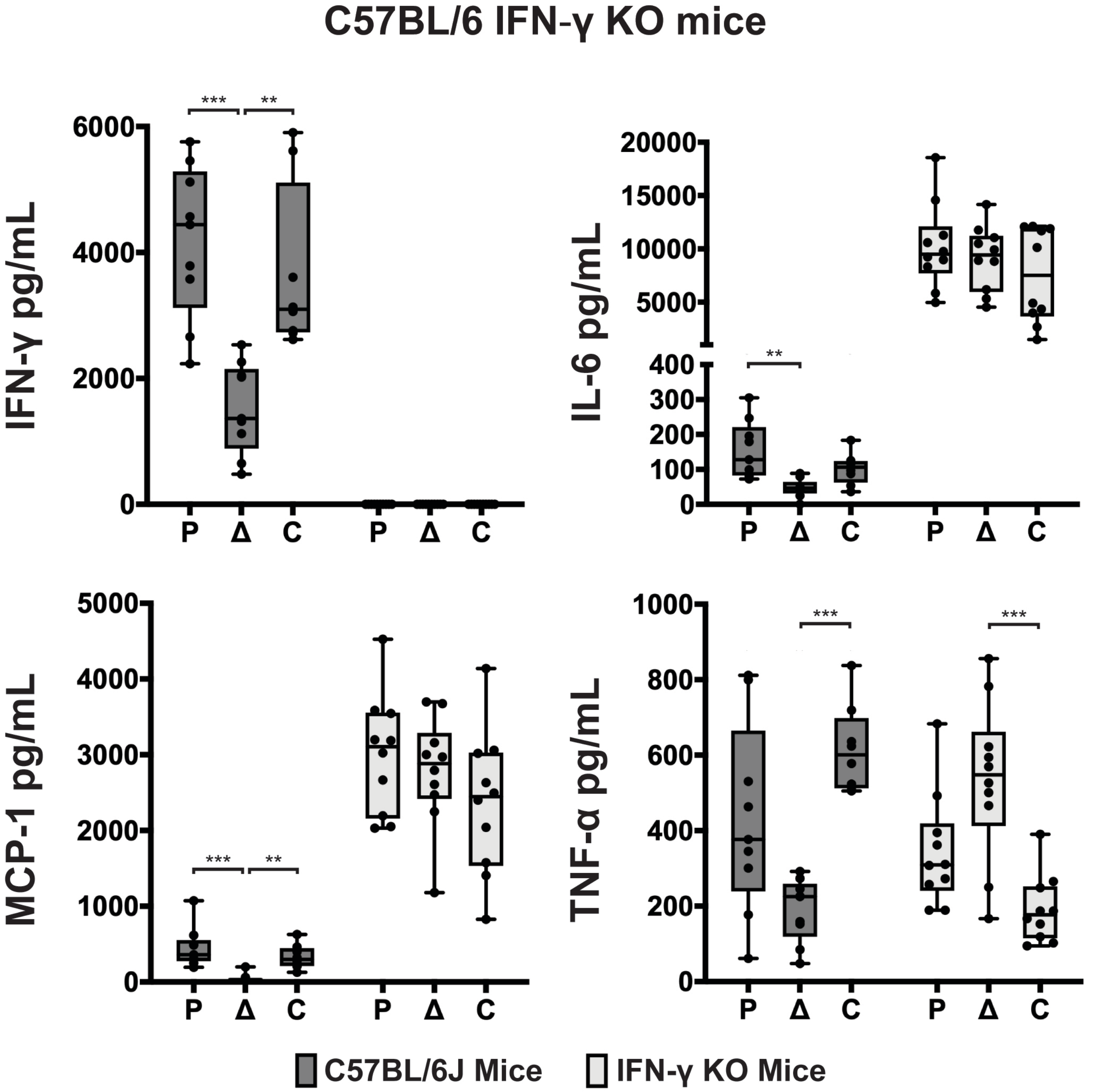

Figure S5. MCP-1 and IL-6 are upregulated in the serum of IFN-γ KO mice during acute infection. Graphs show a comparative profile of the cytokine response in wild-type and IFN-γ KO mice. Blood serum of female and male C57BL/6-WT and IFN-γ KO mice were collected to analyze the cytokine response. Mice were infected with $1x10^4$ parasites of each strain and analyzed at 7 days postinfection. MCP-1 and IL-6 are more abundant in IFN-γ KO mice, but there are no significant differences between strains in IFN-γ KO mice. TNF-α is significantly lower in ΔTgLOX1-infected mice compared to parental- and complement-infected WT mice, but TNF-α is significantly higher in ΔTgLOX1-infected mice compared to parental- and complement-infected IFN-γ KO mice. IL-10 and IL-12p70 were not detected in either mouse strain. The statistics were performed using one way ANOVA in the GraphPad prism software. The *p* value was considered as follows: ** <0.005 and *** <0.0005.
