# Supplementary material for: A Toxoplasma gondii lipoxygenase-like enzyme is necessary for virulence and changes localization associated with the host immune response
Source: mBio. 2023 Aug 30;14(5):e01279-23. doi: 10.1128/mbio.01279-23 (PMC10653942; doi:10.1128/mbio.01279-23)
Supplement: All Legends — Legends for the six supplemental figures and three supplemental movies. [file mbio.01279-23-s0006.docx]

**Figure S1. Generation of the knockout strain ΔTgLOXL1 and its growth in tissue culture.** (A) The homology region for the knockout was designed to flank the upstream and downstream regions of the TgLOXL1 gene. Amplification of the TgLOXL1 gene in wild type (parental) strain showed a 3361 bp band in contrast to the 2879 bp in the ΔTgLOXL1, indicating the loss of the gene. (B) Triplicate monolayers of HFFs were infected with *T. gondii*. At 12, 18, or 36 hours post-infection, tachyzoites per vacuole were scored on blinded slides for at least 100 random vacuoles per replicate. Average tachyzoites per vacuole are shown at each time point. (C) To see the formation of plaques, HFF monolayers were infected with 500 parasites of each strain. After 7 days post-infection, cells were stained with crystal violet to reveal *T. gondii*plaque formation. (D) Shown the number of plaques formed and quantified using ImageJ software. The statistics were performed using one way ANOVA in the GraphPad Prism software. The *p* value was considered as follows: * <0.05 and ** <0.005.

**Figure S2. TgLOXL1 is localized in the cytoplasm of intracellular tachyzoites.** (A) Colocalization of MIC2 (red) and TgLOXL1 (green). TgPL1-HA was a positive control (green) and untagged parasites as a negative control. (B) Colocalization of GRA2, GRA3 and GRA4 and TgLOXL1 (green) in intracellular tachyzoites. All the images were taken at 24 hours post-infection and visualized by epifluorescence microscopy. Nuclei were counterstained with DAPI. Differential Interference Contrast (DIC). Images were taken under the same magnification. All the scale bars are equal to 5 μm.

**Figure S3. ΔTgLOXL1 parasites have reduced parasitemia during chronic infection.** (A) NMRI mice were infected with 1x10^4^ parasites of each strain and at 28 days post-infection, the brains were removed for luciferase assays. The statistics were performed using one way ANOVA in the GraphPad Prism software. The *p* value was considered as <0.005. (B) Survival curve of NMRI mice infected with 1x10^5^ parasites of each strain. Mice infected with parental and complement parasites became moribund between days 9-15. (C) A few cysts were found in the brains of ΔTgLOXL1-infected NMRI mice at 28 days post-infection. Brains were stained for DBA (red) and imaged with Differential Interference Contrast (DIC). Scale bar equals 5 μm. (D) Shown are Swiss Webster mice infected with 1x10^4^ parasites of each strain and at 28 days post-infection, the brains were removed for luciferase assays. (E) Survival curve of 3-5 C57BL/6-WT mice. Shown a combination of two independent experiments of 3-5 mice, either all males or all females, with a total of 6-9 mice per strain. Mice were i.p. infected with 1x10^4^ of each strain and health monitored up to 28 days post-infection. The statistics were performed using one way ANOVA in the GraphPad Prism software. The *p* value was considered as * <0.05.

**Figure S4. ΔTgLOXL1 parasitemia is reduced even with large inoculums.** Shown two independent experiments of 3-5 C57BL/6-WT mice, either all males or all females, with a total of 7-8 mice per strain. Mice were i.p. infected with 1x10^6^ of luciferase-expressing parasites of each strain and 3 days post-infection they were imaged ventrally by IVIS. (A and B) Shown are the images for each strain and separately for gender. For all mice, the abdominal hair was removed to avoid signal interference and the exposure time was the same. (C) Shown the total flux was obtained by measuring the luminescence intensity in the peritoneal cavity of mice. The statistics were performed using one way ANOVA in the GraphPad prism software. The *p* value was considered as ** <0.005.

**Figure S5. MCP-1 and IL-6 are upregulated in the serum of IFN-γ KO mice during acute infection.** Graphs show a comparative profile of the cytokine response in wild-type and IFN-γ KO mice. Blood serum of female and male C57BL/6-WT and IFN-γ KO mice were collected to analyze the cytokine response. Mice were infected with 1x10^4^ parasites of each strain and analyzed at 7 days post-infection. MCP-1 and IL-6 are more abundant in IFN-γ KO mice, but there are no significant differences between strains in IFN-γ KO mice. TNF-α is significantly lower in ΔTgLOXL1-infected mice compared to parental- and complement-infected WT mice, but TNF-α is significantly higher in ΔTgLOXL1-infected mice compared to parental- and complement-infected IFN-γ KO mice. IL-10 and IL-12p70 were not detected in either mouse strain. The statistics were performed using one way ANOVA in the GraphPad prism software. The *p* value was considered as follows: ** <0.005 and *** <0.0005.

**Figure S6. Intracellular proliferation of *T. gondii* in BMDMs.** (A) Shown a representative uninfected BMDMs either naïve or stimulated with 100 ng/ml LPS, 100 U/mL IFN-γ, or 100 U/mL IFN-γ and 100 ng/ml LPS, then imaged after 48 hours**.** (B) Shown a representative infected with untagged parasites and activated BMDMs in cell culture. BMDMs were stimulated with 100 ng/ml LPS, 100 U/mL IFN-γ, or 100 U/mL IFN-γ and 100 ng/ml LPS and imaged after 48 hours poststimulation**.** Naïve BMDMs were used as control. (C) Shown a representative infected with HA-tagged parasites and activated BMDMs in cell culture. BMDMs were stimulated with 100 U/mL IFN-γ and 100 ng/ml LPS, fixed after 24 hours poststimulation and permeabilized with saponin or triton x-100**.**  Parasites were stained for SAG1 (red), TgLOXL1-HA (green) and nuclei were counterstained with DAPI. Differential Interference Contrast (DIC). All scale bars are equal to 10 μm for A panel and 5 µm for B and C panels. (D and E) Shown parasite proliferation of the parental, ΔTgLOXL1 and untagged parasites in BMDMs. BMDMs were infected and then either left naïve or stimulated with 100 ng/ml LPS, 100 U/mL IFN-γ, or 100 U/mL IFN-γ and 100 ng/ml LPS and imaged after 48 hours poststimulation in the IncuCyte. *T. gondii* signal in infected BMDMs was measured by the total fluorescence of SAG1. Dots represent the *T. gondii* signal of SAG1 in individual pictures. Replicate one is shown in panel (D) and replicate 2 is shown in panel (E). Naïve BMDMs were used as control. (F) Shown intracellular replication of parental, ΔTgLOXL1 in stimulated BMDMs under different concentrations of IFN-γ and/or LPS for 48 h. (G) Serum from female and male C57BL/6-WT mice was collected to analyze the cytokine response after meloxicam treatment. Mice were pretreated with 10 mg/kg meloxicam, and either left uninfected or infected with 1x10^4^ parasites of each strain after 24 hours, treated daily, and analyzed at 7 days post-infection. The statistics were performed using one way ANOVA in the GraphPad Prism software. The *p* value was considered as follows: * <0.05 and ** <0.005.

**Movie 1. TgLOXL1 localized in the cytoplasm of intracellular tachyzoites.** 3D projections of parasites expressing HA-tag in the N-terminus TgLOXL1 (green) and the parasite surface localization of SAG1 (red) in intracellular tachyzoites at 24 hours post-infection and visualized by confocal microscopy.

**Movie 2. TgLOXL1 is released by the parasite inside infected leukocytes.** 3D projections of infected leukocytes isolated from the peritoneal cavity of infected mice. Mice were infected with 2x10^6^ N-terminal HA-tag parasites and 3 days post-infection leukocytes were isolated. Parasites were stained for SAG1 (red), TgLOXL1-HA (green) and nuclei were counterstained with DAPI.

**Movie 3. TgLOXL1 is released by the parasite inside infected leukocytes.** 3D projections of infected leukocytes isolated from the peritoneal cavity of infected mice. Mice were infected with 2x10^6^ N-terminal HA-tag parasites and 3 days post-infection leukocytes were isolated. Parasites were stained for SAG1 (red), TgLOXL1-HA (green) and nuclei were counterstained with DAPI.
